# Costs and Cost-Effectiveness of Hypertension Screening and Treatment in Adults with Hypertension in Rural Nigeria in the Context of a Health Insurance Program

**DOI:** 10.1371/journal.pone.0157925

**Published:** 2016-06-27

**Authors:** Nicole T. A. Rosendaal, Marleen E. Hendriks, Mark D. Verhagen, Oladimeji A. Bolarinwa, Emmanuel O. Sanya, Philip M. Kolo, Peju Adenusi, Kayode Agbede, Diederik van Eck, Siok Swan Tan, Tanimola M. Akande, William Redekop, Constance Schultsz, Gabriela B. Gomez

**Affiliations:** 1 Department of Global Health, Academic Medical Center, University of Amsterdam, Amsterdam Institute for Global Health and Development, Pietersbergweg 17, Amsterdam, 1105 BM, The Netherlands; 2 Office of Public Health Studies, University of Hawaii, John A. Burns School of Medicine, 1960 East-West Road, Honolulu, HI, United States of America; 3 Department of Epidemiology and Community Health, University of Ilorin Teaching Hospital, P.M.B. 1459, Ilorin, postal code 240001, Nigeria; 4 Department of Medicine, University of Ilorin Teaching Hospital, P.M.B. 1459, Ilorin, postal code 240001, Nigeria; 5 Hygeia Nigeria Ltd, 13B Idejo Street, Victoria Island, Lagos, Nigeria; 6 Ogo Oluwa Hospital, Bacita, Kwara State, Nigeria; 7 PharmAccess Foundation, Amsterdam, The Netherlands; 8 Institute for Medical Technology Assessment, Erasmus University Rotterdam, Rotterdam, the Netherlands; 9 Department of Global Health and Development, London School of Hygiene and Tropical Medicine, London, United Kingdom; London School of Hygiene and Tropical Medicine, UNITED KINGDOM

## Abstract

**Background:**

High blood pressure is a leading risk factor for death and disability in sub-Saharan Africa (SSA). We evaluated the costs and cost-effectiveness of hypertension care provided within the Kwara State Health Insurance (KSHI) program in rural Nigeria.

**Methods:**

A Markov model was developed to assess the costs and cost-effectiveness of population-level hypertension screening and subsequent antihypertensive treatment for the population at-risk of cardiovascular disease (CVD) within the KSHI program. The primary outcome was the incremental cost per disability-adjusted life year (DALY) averted in the KSHI scenario compared to no access to hypertension care. We used setting-specific and empirically-collected data to inform the model. We defined two strategies to assess eligibility for antihypertensive treatment based on 1) presence of hypertension grade 1 and 10-year CVD risk of >20%, or grade 2 hypertension irrespective of 10-year CVD risk (hypertension and risk based strategy) and 2) presence of hypertension in combination with a CVD risk of >20% (risk based strategy). We generated 95% confidence intervals around the primary outcome through probabilistic sensitivity analysis. We conducted one-way sensitivity analyses across key model parameters and assessed the sensitivity of our results to the performance of the reference scenario.

**Results:**

Screening and treatment for hypertension was potentially cost-effective but the results were sensitive to changes in underlying assumptions with a wide range of uncertainty. The incremental cost-effectiveness ratio for the first and second strategy respectively ranged from US$ 1,406 to US$ 7,815 and US$ 732 to US$ 2,959 per DALY averted, depending on the assumptions on risk reduction after treatment and compared to no access to antihypertensive treatment.

**Conclusions:**

Hypertension care within a subsidized private health insurance program may be cost-effective in rural Nigeria and public-private partnerships such as the KSHI program may provide opportunities to finance CVD prevention care in SSA.

## Introduction

Raised blood pressure is the leading risk factor for disease burden and mortality worldwide, mainly due to associated cardiovascular diseases (CVD).[1,2] Nearly 80% of CVD-related mortality occurs in low- and middle-income countries (LMICs).[3,4] People in LMICs die from CVD at a younger age compared to people in high income countries, often in their most productive years. CVD in LMICs have a large economic impact, both at household and macro-economic level, due to catastrophic healthcare expenditures and through loss of income and labour productivity.[3,4]

The prevalence of hypertension and its complications is increasing rapidly in sub-Saharan Africa (SSA) with an age-standardized hypertension prevalence of 19.1% in 1990 compared to 25.9% in 2010.[1,5,6] Adequate treatment of hypertension greatly reduces the risk of CVD.[7] However, treatment coverage of antihypertensive medication is low due to limited awareness, accessibility and affordability of quality treatment for hypertension in settings with overburdened health systems.[6,8–11] There is an urgent need to develop and evaluate the costs and effects of innovative service delivery models for the management of hypertension that guarantee access to high quality care for patients.

The Kwara State Health Insurance (KSHI) program is an initiative of the Kwara State Government[12], Hygeia Community Health Care (HCHC)[13], the Health Insurance Fund[14] and PharmAccess Foundation[15] to improve access to affordable and quality healthcare for low income population in Kwara State, rural Nigeria. The insurance program provides coverage for primary and limited secondary care, including antihypertensive treatment. In addition, the program aims to improve the quality of care in the participating healthcare facilities by facilitating the upgrade of their infrastructure, training of staff in guideline-based care, and management support. Individual enrolment in the program is voluntary and participants pay about 12% of the premium. At the time of the study, the participant part of the premium was approximately US$ 2 per year, currently the participants pay approximately US$ 3 per year. The remaining part of the premium is subsidized, mainly by the Kwara State Government (see section B in S1 File for more information).

Previous studies have shown hypertension treatment to be a cost-effective intervention in high risk individuals in modelling studies from SSA. [16–21] We previously showed that hypertension management through the KSHI program was effective in reducing blood pressure in a cohort of people with hypertension[22,23] and evaluated costs of hypertension care from a healthcare perspective.[24] In this paper, we aim to evaluate the costs and cost-effectiveness of hypertension management through the KSHI program at scale, for the population at risk of CVD using empirical data from our previous studies and program monitoring databases.

To the best of our knowledge, this study is the first economic evaluation of hypertension treatment in SSA to use empirically collected data on population risk distributions, cost of care, treatment coverage and blood pressure reduction after treatment. In addition, it is the first study to incorporate the costs of setting up and managing a service delivery model that provides access to care for patients. Furthermore, we have tested a large set of assumptions across key model parameters, some of which have not yet been addressed in previous cost-effectiveness studies of hypertension treatment in SSA.[16,18,19,21]

## Materials and Methods

We developed a Markov model to assess the costs and cost-effectiveness of population-level hypertension screening and subsequent antihypertensive treatment in the context of a health insurance program, from the healthcare provider perspective. We compared this intervention to a reference scenario where the insurance program is not operational and people do not have access to screening or treatment for hypertension. The choice for this reference scenario was based on the observation that insurance coverage and antihypertensive treatment coverage in the population was 0% and 4.6% respectively before the program was rolled-out.[22,23] Information regarding the quality of the antihypertensive treatment or the continuity of the treatment was unavailable. We simulated a cohort of 10,000 individuals aged 30–79 years with no previous CVD for ten years. The primary outcome was the incremental cost per disability-adjusted life year (DALY) averted.

### 2.1 Model

We characterized the individuals into 192 unique CVD risk profiles based on sex, age, blood pressure, the presence of diabetes mellitus, smoking status, and total cholesterol. The distribution of risk factors in the population were sourced from population-based household surveys undertaken as part of the overall evaluation of the KSHI program (Table 1). These surveys were conducted in rural Kwara State. In the surveys, demographic, socioeconomic and medical information including measurements of blood pressure, and cholesterol were collected. Ethical clearance for the surveys conducted in Kwara was obtained from the ethical review committee of the University of Ilorin Teaching Hospital (04/08/2008, UITH/CAT189/11/782). Informed consent was obtained from all participants by signature or by fingerprint, as approved by the ethical review committee. The methods used for these surveys and a description of the study setting are described briefly in the supplement (S1 File sections A and B) and in more detail elsewhere. [8,22,23]

**Table 1:** Input parameters for cost-effectiveness analyses.

**Population and risk factor distributions**				
	**Proportion (SE)**	**Average (SE)**	**Distribution**	**Source #**
**Age categories**				
30–44 years old	0.37 (0.01)	35.8 (0.15)	Beta	Kwara HH survey
45–59 years old	0.34 (0.01)	50.1 (0.15)	Beta	Kwara HH survey
60–69 years old	0.19 (0.01)	62.5 (0.14)	Beta	Kwara HH survey
70–79 years old	0.11 (0.01)	71.8 (0.17)	Beta	Kwara HH survey
**Gender, male**	0.45 (0.01)	-	Beta	Kwara HH survey
**Hypertension severity^**				
No hypertension	0.77 (0.01)	114.0 (0.30)	Beta	Kwara HH survey
Hypertension, stage 1	0.13 (0.01)	142.66 (0.56)	Beta	Kwara HH survey
Hypertension, stage 2	0.11 (0.01)	173.49 (1.36)	Beta	Kwara HH survey
**Total Cholesterol**				
TC > 5 mmol/L	0.08 (0.01)	5.49 (0.05)	Beta	Kwara HH survey
TC < = 5 mmol/L	0.92 (0.01)	3.66 (0.02)	Beta	Kwara HH survey
**High Density Lipoprotein Cholesterol**				
TC > 5 mmol/L*	0.08 (0.01)	1.36 (0.09)	Beta	Kwara HH survey
TC < = 5 mmol/L*	0.92 (0.01)	1.08 (0.02)	Beta	Kwara HH survey
**Current daily smoking**	0.12 (0.01)	N.A.	Beta	Kwara HH survey
**Diabetes**	0.04 (0.01)	N.A.	Beta	Kwara HH survey
**Probabilities and outcomes in model**				
**Stroke event**	**Base Case**	**Range**	**Distribution**	**Source #**
Probability of stroke event	Framingham risk score per risk profile per year			[26]
Probability of stroke to be fatal within one year	0.53	0.50–0.57	Triangular	[30–42]
Survival time if stroke fatal within one year	82.0 days	77.6–89.6 days	Triangular	[30–42]
Survival time if stroke non-fatal within one year	Age- and gender-specific, adapted to Nigeria			[43,44]
**CHD event**	**Base Case**	**Range**	**Distribution**	**Source #**
Probability of CHD event	Framingham risk score per risk profile per year			[25]
Probability of CHD to be fatal within one year	0.30	0.26–0.33	Triangular	[16,45,46]
Survival time if CHD fatal within one year	49.3 days	44.3–61.3 days	Triangular	[16,45,46]
Survival time if CHD non-fatal within one year	Age- and gender-specific, adapted to Nigeria			[44,47]
**Other death**			**Distribution**	**Source #**
Probability of non-CVD mortality per year	Age- and gender-specific table in supplement			[44]
**Hypertension treatment**	**Base Case**	**Range**	**Distribution**	**Source #**
Coverage in KSHI program	29%	-	-	Kwara HH survey
SBP decrease–individuals on antihypertensive treatment (mmHg)	-20	(-31.6–-8.4)	Triangular	Kwara HH survey
SBP decrease–screened hypertensive individuals, not on antihypertensive treatment (mmHg)	-2.4	(-6.0–0)	Triangular	Kwara HH survey
**Relative risk reduction (RRR) per 10 mmHg SBP decrease**	**Base Case**	**Range**	**Distribution**	**Source #**
RRR Stroke–based on Lawes 30–44 years old	2.38	2.13–2.63	Triangular	[7]
RRR Stroke–based on Lawes 45–59 years old	2	1.92–2.04	Triangular	[7]
RRR Stroke–based on Lawes 60–69 years old	1.56	1.52–1.61	Triangular	[7]
RRR Stroke–based on Lawes 70–79 years old	1.37	1.32–1.43	Triangular	[7]
RRR CHD–based on Lawes 30–44 years old	1.92	1.54–2.38	Triangular	[7]
RRR CHD–based on Lawes 45–59 years old	1.67	1.56–1.75	Triangular	[7]
RRR CHD–based on Lawes 60–69 years old	1.33	1.27–1.39	Triangular	[7]
RRR CHD–based on Lawes 70–79 years old	1.25	1.191.32	Triangular	[7]
RRR Stroke–based on Rapsomaniki	1.16	1.14–1.18	Triangular	Calculated from[48]
RRR CHD–based on Rapsomaniki	1.16	1.15–1.18	Triangular	Calculated from[48]
**Cost parameters (2012 US$)**				
	**Base Case**	**Range**	**Distribution**	**Source #**
Cost of hypertension care per patient per year	112	101–126	Triangular	Adapted from [24]
Cost of screening per person screened	5	4–6	Triangular	[49]
Above-service delivery costs of insurance program management per patient per year	24	-	Triangular	KSHI program management
Cost of acute care after a stroke per patient	380	242–1,556	Triangular	Base Case: UITH data, [24] Range: [16,17,19,35,50–57]
Cost of follow up care after a stroke per patient per year	240	206–275	Triangular	[24]
Cost of acute care after CHD event per patient	181	115–1,180	Triangular	Base Case: UITH data, [24] Range: [16,17,19]
Cost of follow up care after CHD event per patient per year	278	235–320	Triangular	[24]
**DALY assumptions**				
	**Base Case**	**Range**	**Distribution**	
Disability weight during survival period after a fatal stroke (death during first year)	0.553	0.363–0.738	Triangular	Adapted from [27]
Disability weight during survival after a non-fatal stroke	0.256	0.021–0.553	Triangular	Adapted from [27]
Disability weight during survival period after a fatal CHD event (death during first year)	0.180	0.135–0.250	Triangular	Adapted from [27]
Disability weight during survival after a non-fatal CHD event	0.09	0.022–0.234	Triangular	Adapted from [27]
Disability weight while on antihypertensive treatment	0.031	0.017–0.05	Triangular	[27]

# Details on assumptions made and sources used can be found in the supplemental material.

^ No hypertension: SBP < 140 AND DBP < 90; Hypertension grade 1: SBP between 140–159 mmHg and/or DBP between 90–99 mmHg; Hypertension grade 2: SBP of at least 160 mmHg and/or DBP of at least 100 mmHg.

* HDL was not taken as a separate variable for defining risk profiles. HDL average was calculated for high and low TC groups.

Abbreviations: SE: Standard error; Kwara HH survey: Kwara household survey; TC: Total cholesterol; CHD: Coronary heart disease; CVD: Cardiovascular disease; SBP: Systolic blood pressure; DBP: Diastolic blood pressure; RRR: Relative risk reduction; KSHI: Kwara State Health Insurance; UITH: University of Ilorin Teaching Hospital; DALY: Disability adjusted life year.

**Fig 1:**
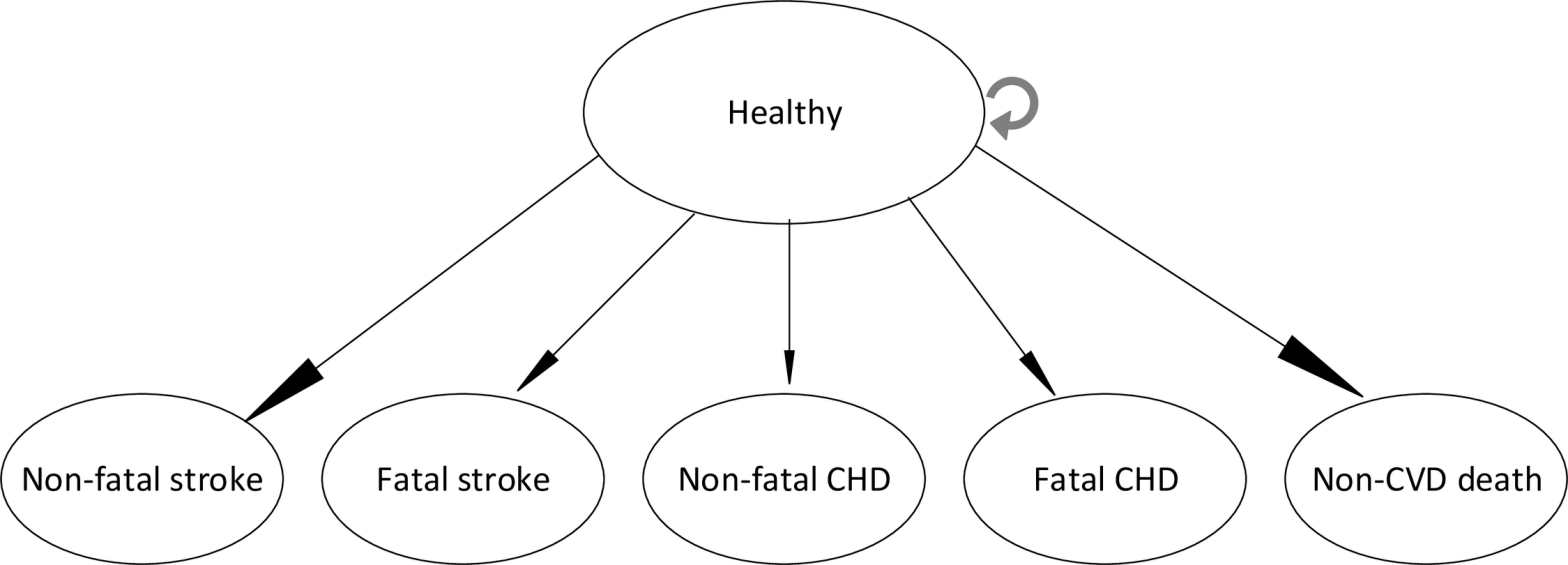
Structure of the Markov model.

In our model, every year individuals experience a risk of having a stroke or a coronary heart disease (CHD) event; these events could be either fatal (defined as death within one year of the event) or non-fatal (Fig 1).

The probabilities of having a stroke or a CHD event (including myocardial infarction (MI) and angina pectoris (AP)) were based on the Framingham ten-year risk equations for stroke and CHD [25,26] and converted to one-year probabilities for every one-year cycle in the ten-year period. The proportions of fatal and non-fatal stroke and CHD events as well as the average number of days of survival after CVD events that led to death within the first year after the event were based on figures from the literature (Table 1, and Table D, Table E and section E in S1 File). For non-fatal CVD events, we did not model repeat events but assigned an average survival time according to age and gender, based on figures from the literature (Table 1, and Table D, Table E and section G in S1 File). Finally, individuals could also die from non-CVD related causes. Non-CVD mortality was sourced from the 2010 Global Burden of Disease study (Table 1, and Table A and section E in S1 File).[27]

### 2.2 Intervention

The intervention modeled is population-level hypertension screening and subsequent antihypertensive treatment for high CVD risk individuals in the context of the KSHI program. We defined two eligibility strategies for treatment. The first was a CVD risk and hypertension-based strategy, where all individuals with hypertension stage 1 (systolic blood pressure between 140–159 mmHg and/or diastolic blood pressure between 90–99 mmHg)[28] combined with a ten-year CVD risk greater than 20% as well as all individuals with hypertension stage 2 (systolic blood pressure of at least 160 mmHg and/or diastolic blood pressure of at least 100 mmHg) [28], regardless of their ten-year CVD risk, were eligible for hypertensive treatment.[29] These groups are eligible for treatment according to the guidelines used in the KSHI program. The second strategy was CVD risk based, where all hypertensive individuals with a ten-year CVD risk greater than 20% were eligible for treatment. This approach is comparable to strategies tested in previous cost-effectiveness studies.[16,18,19,21] We will refer to the two strategies as the risk and hypertension based strategy and risk based strategy, respectively.

We assumed that the intervention reduced the probability of having a CVD event. Mortality and morbidity after a CVD event were assumed to be equal in the KSHI scenario and the reference scenario (Table 1). Finally, the coverage of the intervention was assumed to be the same as the observed antihypertensive treatment coverage in a population living in an area with access to the KSHI program for four years. As the observed treatment coverage was four years after access to antihypertensive care became available, this estimate includes attrition to the program over this timeframe (Table 1, and section F, S1 File).

### 2.3 Costs

We considered: 1) costs of delivering hypertension care within the context of the KSHI program, and 2) costs of CVD events (including acute care and follow-up care). The costs of delivering hypertension care were assigned only to the intervention scenario while the costs of CVD events were assigned to events in both the intervention and the reference scenario. The costs of delivering hypertension care included population-level screening costs, service delivery for individual hypertension treatment and insurance program costs associated with the local operations of HCHC and program management at PharmAccess level, that we will refer to as above-service delivery costs. The cost of population-level screening was derived from WHO estimates.[49] Service delivery cost for hypertension care was sourced from a costing study in the Ogo Oluwa Hospital, a private hospital participating in the KSHI program.[24] These costs included both direct costs for consultation, tests and drugs as well as indirect building and overhead costs. We included costs of antihypertensive drugs and acetyl salicylic acid (aspirin) based on observed utilization in Ogo Oluwa Hospital. An overview of components can be found in Table F and section H in S1 File. More details on the approach and methodology of the costing study can be found in the original publication.[24] Above-service delivery costs were added as a mark-up to all individuals on antihypertensive treatment (Table 1). The calculation of the above-service delivery costs was based on realized and projected expenses and is explained in detail in S1 File, Table H and section H.

Using an ingredients approach, we estimated acute care costs for stroke based on guidelines (from LMIC for stroke[58–60] and in absence of LMIC guidelines from HIC for CHD[61,62]) as well as discussion with local specialists from the University of Ilorin Teaching Hospital (UITH; a tertiary hospital in the program area which is one of the referral hospitals for patients in the KSHI program) to determine what was feasible and available in the setting of our study. Acute care costs included in-hospital stay, tests, and drugs. Highly specialized clinical interventions were excluded. Data from the costing study as well as additional data from UITH on utilization and costs were used (Table 1, and Table I and section H, S1 File).

We validated acute care costs with figures from WHO-CHOICE, National Health Interview Survey (NHIS) and literature from SSA, excluding data from South Africa where the standard of care is higher compared to the rest of SSA.[16,17,19,35,50–57] Costs for follow-up care after CVD events were assumed to be equal to antihypertensive treatment costs with the addition of a number of drugs, depending on the event (Table 1, and Table J and section H, S1 File). All prices were collected in local currency and are presented in 2012 US$ using the mean exchange rate of the study period (1 US$ = 154.4 NGN). Cost estimates derived from other studies were adjusted for inflation using standard methods.[63]

### 2.4 Analysis

The intervention effect was attributed to two components: 1) screening for hypertension combined with lifestyle advice for individuals with high blood pressure, and 2) antihypertensive drug treatment. The magnitude of the effect was based on the results of an impact study that we conducted in the program area where people were screened for hypertension, offered insurance and access to hypertension treatment.[23] We applied blood pressure reductions as observed for 1) individuals who were screened, told to have hypertension and given information about lifestyle measures but who were not on pharmacological treatment during the four year follow-up survey that was part of the impact study; and 2) for individuals who were also treated with antihypertensive drugs (Table 1, and section F, S1 File).

Subsequently, reductions in blood pressure were translated into reductions of CVD risk which were applied to the annual risks of stroke and CHD events. For this step, we tested three different assumptions in scenarios. First, we used the observed blood pressure reduction to recalculate the Framingham scores for each individual risk profile. Additionally, we applied two estimates of relative risk reduction that were derived from meta-analyses on the effect of lowering blood pressure on stroke and CHD outcomes. We compared relative risk reduction based on a recent meta-analysis by Rapsomaniki et al[48], to the relative risk reduction estimate described by Lawes et al.[7,64] We will refer to these scenarios as “Framingham”, “Rapsomaniki”, and “Lawes” assumptions (Table 1, and Table B and section F, S1 File).

Mortality and morbidity outcomes were translated into years of life lost (YLLs) due to premature death and years lived with disability (YLDs), respectively, to calculate the total number of DALYs in each scenario. To calculate YLDs, we applied disability weights due to CVD events based on the utilities defined in the GBD 2010 (Table 1, and Table C and section G, S1 File).[27] We also applied a disability weight to the period of time when patients were on antihypertensive treatment.[27] We used standard methods[65] without age weighting[27] to calculate DALYs. Incremental cost-effectiveness ratios (ICER) were calculated as the difference in costs divided by the difference in DALYs between the intervention and reference scenario. We took a lifetime perspective when calculating costs and health benefits following from events that occurred in the ten-year intervention timeframe. Future costs and health outcomes were discounted at 3% per year.

### 2.5 Uncertainty and sensitivity analysis

We constructed 95% confidence intervals around the primary outcome results through a probabilistic sensitivity analysis (PSA; Monte Carlo simulation) in which parameters were randomly sampled from their probability distributions in 1,000 iterations (Table 1). Results from the PSA are presented in cost-effectiveness acceptability curves.[66] Additionally, one-way sensitivity analyses were conducted in which model parameters were varied outside their confidence intervals. We tested the sensitivity of our model to the proportion of stroke and CHD events that were fatal within one year; the length of survival of non-fatal events; variation in above-service delivery costs; the costs of acute care after a stroke event; the costs of acute care after a CHD event; costs of hypertension care; a change in effect of blood pressure treatment; increased CVD risk at baseline; and discount rates.

The model was programmed using Microsoft Excel 2013 (Microsoft Corp), population distribution and effectiveness data were calculated using Stata (version 12.0; StataCorp). We conducted and present this study following the Consolidated Health Economic Evaluation Reporting Standards (CHEERS) guidelines[67] and the published standards of the Bill and Melinda Gates Foundation, Methods for Economic Evaluation Project.[68]

## Results

### 3.1 Health impact

Population-level screening and providing anti-hypertensive treatment in the context of a voluntary health insurance program using the risk and hypertension based strategy would result in a 4%, 3%, and 8% reduction in the number of stroke events and a 2%, 3% and 6% reduction in the number of CHD events using the Framingham, Rapsomaniki, and Lawes assumptions respectively. In the risk based strategy, a lower number of stroke and CHD events would be avoided (3% and 2% respectively for the Framingham assumption, 3% and 2% for the Rapsomaniki assumption and 7% and 4% for the Lawes assumption). The total number of DALYs averted in a population of 10,000 individuals are presented in Tables 2 and 3 by treatment eligibility strategy.

**Table 2:** Outcomes in costs (US$ 2012) and DALYs averted for a cohort of 10,000 individuals.

	Strategy 1. Treatment eligibility: risk and hypertension based				Strategy 2. Treatment eligibility: risk based			
	Reference scenario	KSHI Framing-ham	KSHI Rapso-maniki	KSHI Lawes	Reference scenario	KSHI Framing-ham	KSHI Rapso-maniki	KSHI Lawes
Stroke events	241	232	233	221	241	234	235	225
CHD events	416	408	404	392	416	410	407	398
DALYs averted (from events prevented)	reference	130	142	342	reference	95	106	259
DALYs lost (due to treatment)	reference	80	80	81	reference	33	33	34
NET DALYs averted	reference	50	62	261	reference	62	73	226
**Total costs**	607,608	996,082	995,255	973,734	607,608	790,766	789,974	772,617
Screening Costs	0	47,400	47,400	47,400	0	47,400	47,400	47,400
Hypertension treatment costs	0	293,674	293,893	296,196	0	121,395	121,595	122,775
Above-service delivery costs for KSHI program	0	63,453	63,500	63,998	0	26,229	26,273	26,527
Care after CVD event costs	607,608	591,555	590,462	566,141	607,608	595,741	594,707	575,915
Reduction in acute and follow-up costs after CVD event	reference	2.6%	2.8%	6.8%	reference	2.0%	2.1%	5.2%
% of total costs related to KSHI intervention*	0%	36%	36%	36%	0%	25%	25%	27%

*Total costs related to KSHI intervention includes screening, hypertension treatment and above-service delivery costs. Framingham: assuming recalculation of Framingham equation; Rapsomaniki: assuming relative risk reduction based on Rapsomaniki[48]; Lawes: assuming relative risk reduction based on Lawes[7] Abbreviations: KSHI: Kwara State Health Insurance; CHD: Coronary Heart Disease; DALYs: Disability Adjusted Life Years; CVD: Cardiovascular disease.

**Table 3:** Cost-effectiveness of KSHI program (US$ 2012).

	Strategy 1. Treatment eligibility: risk and hypertension based					Strategy 2. Treatment eligibility: risk based				
	Total cost incurred	Total DALYs incurred	DALYs averted	ICER compared to SoC, mean	ICER compared to SoC, MC, median (2.5–97.5 percentile)	Total cost incurred	Total DALYs incurred	DALYs averted	ICER compared to SoC, mean	ICER compared to SoC, MC, median (2.5–97.5 percentile)
**Reference scenario**	607,608	5,086	ref	ref	ref	607,608	5,086	ref	ref	ref
**KSHI Framing- ham**	996,082	5,036	50	7,815	6,282 (dominated to 48,193)	790,766	5,024	62	2,959	2,644 (1,270 to 14,379)
**KSHI Rapso-maniki**	995,255	5,024	62	6,256	5,315 (dominated to 45,211)	789,974	5,013	73	2,498	2,221 (1,121 to 8,484)
**KSHI Lawes**	973,734	4,826	260	1,406	1,287 (dominated to 3,317)	772,617	4,860	226	732	634 (306 to 2,021)

Framingham: assuming recalculation of Framingham equation; Rapsomaniki: assuming relative risk reduction based on Rapsomaniki[48]; Lawes: assuming relative risk reduction based on Lawes[7] Abbreviations: KSHI: Kwara State Health Insurance; DALYs: Disability Adjusted Life Years; ICER: incremental cost-effectiveness ratio; SoC: standard of care. MC: monte carlo simulation.

### 3.2 Costs

The cost per individual in the reference scenario was US$ 60.8, corresponding to acute event care and secondary prevention. The cost per individual of the risk and hypertension based strategy was US$ 99.6 for the Framingham, US$ 99.5 for the Rapsomaniki and US$ 97.4 for the Lawes scenario. The cost per individual of the risk based strategy was lower compared to the risk and hypertension based strategy and was estimated at US$ 79.1 for the Framingham, US$ 79.0 for the Rapsomaniki and US$ 77.3 for the Lawes scenario (Table 3). The reduction in costs attributable to a reduction in costs for acute care of CVD events and follow up care due to the intervention was between 2% and 7%, depending on the scenario (Table 2).

### 3.3 Cost-effectiveness

The mean ICERs for the risk and hypertension based strategy were US$ 7,815 using the Framingham assumption, US$ 6,256 using the Rapsomaniki assumption and US$ 1,406 using the Lawes assumption. The mean ICERs for the risk based strategy were substantially lower at US$ 2,959, US$ 2,498 and US$ 732 using the Framingham, Rapsomaniki and Lawes assumptions respectively (Table 3). The uncertainty bounds around these estimates are presented in Table 3.

The mean ICER in Lawes for the risk and hypertension based strategy and the mean ICER in Lawes and Rapsomaniki in the risk based strategy are considered cost-effective at a willingness-to-pay threshold of one GDP per capita per DALY averted, which was US$ 2,742 in Nigeria in 2012.[69] However, only one set of assumptions could consistently be considered cost-effective when we include uncertainty in model parameters (risk based strategy, Lawes).

### 3.4 Uncertainty analysis

**Fig 2:**
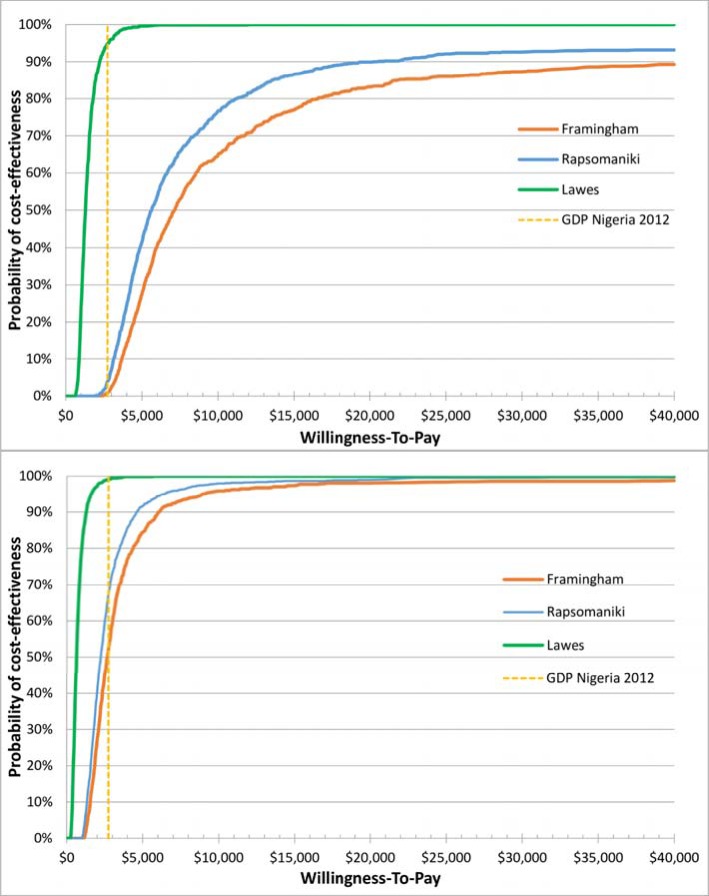
**2A: Cost-effectiveness acceptability curve, risk and hypertension based strategy. Fig 2B: Cost-effectiveness acceptability curve, risk based strategy.** Legend Fig 2A and 2B: GDP Nigeria 2012: US$ 2,742; Framingham: assuming recalculation of Framingham equation; Rapsomaniki: assuming relative risk reduction based on Rapsomaniki[48]; Lawes: assuming relative risk reduction based on Lawes[7].

Fig 2A and 2B show the result of the probabilistic sensitivity analysis and illustrate the probability of antihypertensive treatment provided through the KSHI program to be cost-effective against a range of willingness-to-pay thresholds. At a willingness-to-pay threshold of US$ 2,742 (one GDP per capita), the probability of the risk and hypertension based strategy to be cost-effective compared to the reference scenario was 1%, 4% and 95% respectively under the Framingham, Rapsomaniki and Lawes assumptions (Fig 2A). The probability of the program to be cost-effective when using a risk based strategy was 52%, 68% and 99% respectively under the Framingham, Rapsomaniki and Lawes assumption (Fig 2B).

The bounds of the 95% confidence intervals included negative ICERs. Negative ICERs in the case of our model do not indicate a cost-reduction but indicate cases where DALYs are incurred rather than averted following the burden of being on treatment for long periods of time without having any events (the strategy is then considered dominated). This occurred in 7% and 3.9% of the 1,000 iterations for the Framingham and the Rapsomaniki scenarios in the risk and hypertension based strategy and in 0.3% and 0.2% respectively in the risk based strategy. We present the cost-effectiveness plane for all iterations in Fig A, S1 File.

**Fig 3:**
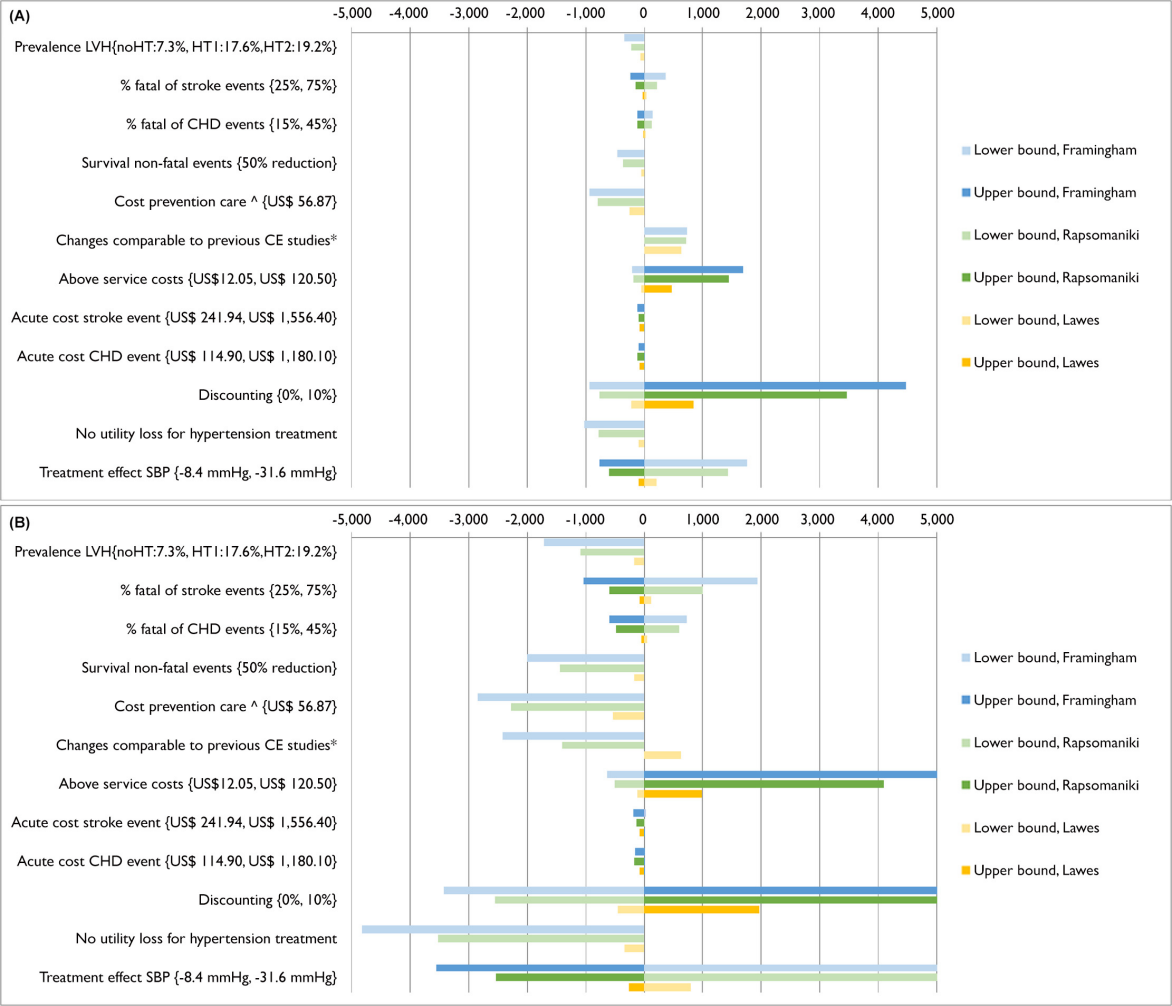
**3A: One-way sensitivity analysis, risk and HT based strategy. Fig 3B: One-way sensitivity analysis, risk based strategy.** Legend Fig 3A and 3B: Presents the change in ICER (incremental costs per DALY averted) compared to the baseline when parameter input is either varied in a high and low bound or when parameter input is varied to an alternative scenario (presented as lower bound). Darker and lighter bars represent the change in ICER when a parameter is varied to a respectively lower value (or alternative scenario) and higher value compared to the baseline estimate. *effect of treatment on SBP: -14.6, coverage of 100% for eligible patients and no disability loss for hypertension treatment. ^based on observed costs in a scenario when limited diagnostic testing and task-shifting from doctors to nurses[24]. Abbreviations: SBP: systolic blood pressure; CHD: coronary heart disease; LVH: left ventricle hypertrophy. noHT: no hypertension; HT1: hypertension stage 1; HT2: hypertension stage 2. All values for the parameters tested as well as resulting ICERs are reported in Tables K and L (S1 File).

One-way sensitivity analyses illustrated that our primary outcome estimates were sensitive to variations in discount rate, effect of treatment on systolic blood pressure, above-service delivery costs, inclusion of disability weights for being on antihypertensive treatment and costs of hypertension treatment (Fig 3A and 3B, and Tables K and L, S1 File. In particular, our estimates based on Framingham and Rapsomaniki assumptions were less robust than those based on Lawes assumptions to variations in key parameters.

## Discussion

Our study found that screening and treatment for hypertension within a health insurance program in Nigeria could be cost-effective at a willingness-to-pay threshold of one GDP per capita per DALY averted. The likelihood of having a cost-effective program was higher when the hypertensive population eligible for treatment was restricted to those with a high 10-year CVD risk, than when eligibility was expanded to the population with grade 2 hypertension, irrespective of 10-year CVD risk.

Our conclusion was sensitive to underlying assumptions and is presented with substantial uncertainty. Our assumption on CVD risk reductions following treatment of high blood pressure had a large effect on the ICERs. When we applied relative risk reductions as reported by WHO (Lawes), between 95% (for the risk and hypertension based strategy) and 99% (for the risk based strategy) of the iterations were considered cost-effective at a willingness-to-pay threshold of one GDP per capita. When we used a recalculation of the Framingham score after treatment to determine risk reduction, only 1% and 52% of the iterations were considered cost-effective respectively.

The reference scenario to which the KSHI scenario is compared represents no access to hypertension care. This is the best estimate of base case currently in our setting. The KSHI scenarios (including two eligibility criteria) present our best representation of the clinical practice expected in a KSHI setting. In the one-way sensitivity analyses, we vary our input parameters to extreme ranges in order to assess the robustness of the findings. A reduction in hypertension care costs increased cost-effectiveness. The estimated reduction in costs was based on a combination of task-shifting from doctors to nurses, minimal target organ damage screening, and a reduction in the number of consultations per year.[24] Studies are needed to determine how and if these changes in service have an effect on health outcomes. An increase in insurance program costs decreased the cost-effectiveness of hypertension care. For the base case insurance program costs, we used a weighted average of realized and projected costs of the first 12 years of the program. This includes costs of starting up and scaling up of the program that are expected to decrease over time. We varied this parameter widely to reflect the possibility of this intervention being set up in another context with much higher program costs. An increase or decrease of systolic blood pressure reduction in response to treatment increased and decreased the cost-effectiveness respectively. However, our base case reflects the systolic blood pressure reduction observed in a population living in an area with access to the KSHI program.

Our findings demonstrate limitations of cost-effectiveness analyses for CVD prevention in SSA. First, there are no long term prospective cohort studies from SSA that have evaluated the association between (change in) CVD risk factors and CVD events and therefore there are no validated equations to determine 10-year CVD risk or relative risk reductions after treatment for populations from SSA. Furthermore, the Framingham equation for stroke is not validated for individuals below 55 years of age and is not intended as a tool to recalculate stroke risk after treatment. Therefore, our use of the Framingham equations might have underestimated the baseline risk of our population as evidence suggests that people from SSA have higher incidence of CVD and CVD-related mortality at a younger age and at lower blood pressure levels compared to people from Caucasian descent.[70–72]

Secondly, the younger age groups with grade 2 hypertension may have a lower 10-year CVD risk but a high lifetime risk, as has been shown in particular for people from African descent.[70–72] Clinical guidelines therefore recommend antihypertensive treatment irrespective of 10-year CVD risk in people with grade 2 hypertension.[29,73] For the younger age groups, the majority of the CVD events is expected to take place after the 10-year time horizon of the intervention. Our model therefore did not capture all the benefits of treatment. At the same time, the younger age groups did incur a disability weight for using antihypertensive treatment during the full time span of the model. This might have led to conservative cost-effectiveness estimates. This also explains why a risk based strategy could be considered more cost-effective, as less (young) people would be eligible for treatment and therefore will not incur the disability weight for antihypertensive treatment. Thirdly, like other studies[16–19,21], we limited our analysis of CVD events to stroke and CHD and did not include other CVD such as heart failure, vascular renal failure, and peripheral artery disease because equations to estimate the probability of events or risk reduction after treatment were not available. However, it is known that especially Africans with hypertension are at increased risk to develop renal and heart failure.[74–78] Therefore, our estimates of cost-effectiveness are likely to be conservative. Fourthly, we did not model subsequent events following a primary event. Unfortunately, there is scarce data from SSA on the probability and costs of re-events, or on disability and survival after subsequent events following a primary event to inform our model. Therefore, we take into account re-events by using average survival time after an event. This average survival includes life years lost due to re-events as well as follow-up care costs by means of secondary prevention of CVD events. Fifthly, by comparing each scenario to the base case separately, we are providing a limited scope of the effects that might be achieved with each intervention. While this scoping exercise is valuable given the paucity of CVD-related economic evaluations in SSA, ideally, we would compare mutually exclusive scenarios simultaneously to identify the optimal expansion pathway given a cost-effectiveness threshold. Finally, we did not estimate the effect of scale on unit costs, for lack of evidence of economies or diseconomies of scale in this population and restricted the analysis to a healthcare provider perspective. As CVD in an African population typically occurs during the productive age[70], a societal perspective taking productivity losses due to CVD into account would probably have resulted in more cost-effective estimates of preventive treatment.

Previous studies evaluating cost-effectiveness of CVD prevention or hypertension care in SSA reported lower ICERs compared to our results.[16–19,21] However, most studies make assumptions on costs and treatment effects based on international databases and studies from high income countries.[16,18,19,21] In general, they use lower estimates for costs of preventive treatment, do not include disability weights for being on antihypertensive treatment (exclusion of this disability weight in a one-way analysis showed an improvement in cost-effectiveness), and use relative risk reductions based on the Lawes study [7] that reports larger effects of treatment compared to other studies.[48] The strength of our study is, therefore, the use of empirically collected data for population risk distributions, costs of care, treatment coverage, blood pressure reduction after treatment, and the use of several sources for CVD risk reduction after treatment to illustrate the uncertainty around these estimates. In line with the recommendations from the global burden of disease study [27], we included a 0.031 disability weight for using antihypertensive treatment as the use of chronic preventive treatment has been shown to reduce the quality of life.[79] This is especially relevant in settings with long travel and waiting times for healthcare such as in SSA.

Recent discussions about willingness-to-pay thresholds have raised the concern that any chosen threshold (such as one or three GDP per capita) is of limited value for decision making as interventions can be cost-effective but not affordable or feasible to implement.[80] This is particularly relevant in our study, as GDP per capita is a national measure, while we aim to inform decision making in one of Nigeria’s poorest states.

In settings such as in Nigeria, where almost 66% of healthcare expenditures is paid out-of-pocket by patients[11], coverage of CVD prevention treatment will remain low if access to care for patients is not guaranteed. Although CVD preventive treatment may be considered cost-effective, implementation is hampered by lack of affordability due to low healthcare budgets in many SSA countries. [10,11] For example, total public healthcare expenditure per capita was only US$ 93 in Nigeria in 2012.[81] We evaluated cost-effectiveness of hypertension care within a health insurance program for low income groups. The co-premium for enrollees of the health insurance program of US$ 3 is low and represents 1% of the annual per capita consumption in the lowest consumption quintile of the population in which the KSHI program is available. However, for some of the poorest households even this small co-premium is still not affordable. To ensure equity and accessibility, the Kwara State Government, which subsidizes the remainder of the premium, has the intention to exempt the poorest vulnerable groups from co-premiums in the future. In addition, other states in Nigeria are aiming for universal coverage in the future. By embedding hypertension care in an operational program and including above-service delivery costs in our analysis, our cost-effectiveness estimates move away from a theoretical analysis and reflect the potential results of a real world intervention that has been proven feasible in providing patients with access to care. Policymakers can compare our results to the cost-effectiveness of CVD prevention care delivery through other programs, thereby making informed choices about the best strategy to combat the increasing burden of CVD.

## Conclusions

Hypertension screening and treatment may be cost-effective in rural Nigeria, at a willingness-to-pay threshold of one GDP per capita per DALY averted, with an important uncertainty around this conclusion. Even if cost-effective, CVD prevention may not be affordable in many SSA settings within current levels of government healthcare expenditures. Public-private partnerships such as the KSHI program provide opportunities to finance CVD prevention care in SSA.

## Supporting Information

S1 File Methods and additional results. (DOCX)
