# Correction: Costs and Cost-Effectiveness of Hypertension Screening and Treatment in Adults with Hypertension in Rural Nigeria in the Context of a Health Insurance Program

**DOI:** 10.1371/journal.pone.0162421

**Published:** 2016-09-12

**Authors:** The PLOS ONE Staff

Within Fig 3, Fig 3A and 3B are incorrectly reversed. The authors have provided the correct Fig 3 here.

**Fig 3:**
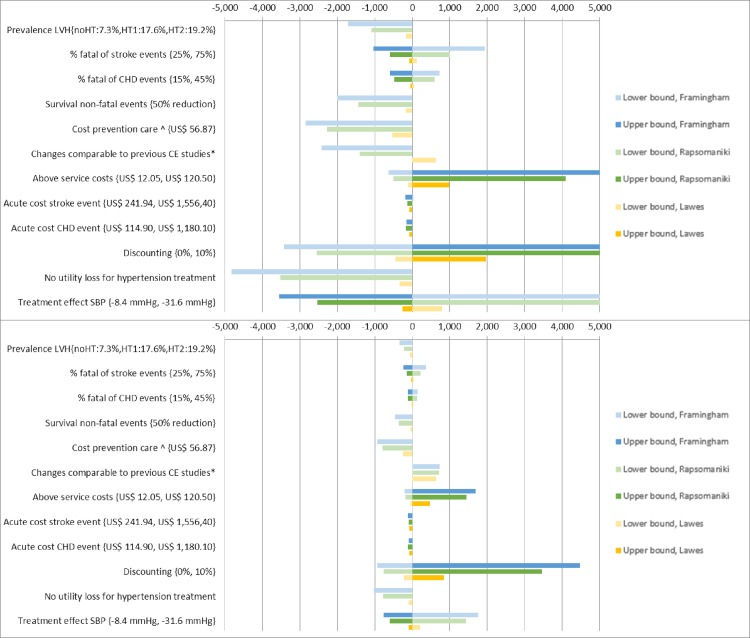
**3A: One-way sensitivity analysis, risk and HT based strategy. Fig 3B: One-way sensitivity analysis, risk based strategy.** Legend Fig 3A and 3B: Presents the change in ICER (incremental costs per DALY averted) compared to the baseline when parameter input is either varied in a high and low bound or when parameter input is varied to an alternative scenario (presented as lower bound). Darker and lighter bars represent the change in ICER when a parameter is varied to a respectively lower value (or alternative scenario) and higher value compared to the baseline estimate. *effect of treatment on SBP: -14.6, coverage of 100% for eligible patients and no disability loss for hypertension treatment. ^based on observed costs in a scenario when limited diagnostic testing and task-shifting from doctors to nurses[24]. Abbreviations: SBP: systolic blood pressure; CHD: coronary heart disease; LVH: left ventricle hypertrophy. noHT: no hypertension; HT1: hypertension stage 1; HT2: hypertension stage 2. All values for the parameters tested as well as resulting ICERs are reported in Tables K and L (S1 File).

There is an error in Table 1. The row “Relative risk reduction (RRR0 per 10 mmHg SBP decrease” was incorrectly omitted. The publisher apologizes for this error.

**Table 1:** Input parameters for cost-effectiveness analyses.

**Population and risk factor distributions**				
	**Proportion (SE)**	**Average (SE)**	**Distribution**	**Source #**
**Age categories**				
30–44 years old	0.37 (0.01)	35.8 (0.15)	Beta	Kwara HH survey
45–59 years old	0.34 (0.01)	50.1 (0.15)	Beta	Kwara HH survey
60–69 years old	0.19 (0.01)	62.5 (0.14)	Beta	Kwara HH survey
70–79 years old	0.11 (0.01)	71.8 (0.17)	Beta	Kwara HH survey
**Gender, male**	0.45 (0.01)	-	Beta	Kwara HH survey
**Hypertension severity^**				
No hypertension	0.77 (0.01)	114.0 (0.30)	Beta	Kwara HH survey
Hypertension, stage 1	0.13 (0.01)	142.66 (0.56)	Beta	Kwara HH survey
Hypertension, stage 2	0.11 (0.01)	173.49 (1.36)	Beta	Kwara HH survey
**Total Cholesterol**				
TC > 5 mmol/L	0.08 (0.01)	5.49 (0.05)	Beta	Kwara HH survey
TC < = 5 mmol/L	0.92 (0.01)	3.66 (0.02)	Beta	Kwara HH survey
**High Density Lipoprotein Cholesterol**				
TC > 5 mmol/L*	0.08 (0.01)	1.36 (0.09)	Beta	Kwara HH survey
TC < = 5 mmol/L*	0.92 (0.01)	1.08 (0.02)	Beta	Kwara HH survey
**Current daily smoking**	0.12 (0.01)	N.A.	Beta	Kwara HH survey
**Diabetes**	0.04 (0.01)	N.A.	Beta	Kwara HH survey
**Probabilities and outcomes in model**				
**Stroke event**	**Base Case**	**Range**	**Distribution**	**Source #**
Probability of stroke event	Framingham risk score per risk profile per year			[26]
Probability of stroke to be fatal within one year	0.53	0.50–0.57	Triangular	[30–42]
Survival time if stroke fatal within one year	82.0 days	77.6–89.6 days	Triangular	[30–42]
Survival time if stroke non-fatal within one year	Age- and gender-specific, adapted to Nigeria			[43,44]
**CHD event**	**Base Case**	**Range**	**Distribution**	**Source #**
Probability of CHD event	Framingham risk score per risk profile per year			[25]
Probability of CHD to be fatal within one year	0.30	0.26–0.33	Triangular	[16,45,46]
Survival time if CHD fatal within one year	49.3 days	44.3–61.3 days	Triangular	[16,45,46]
Survival time if CHD non-fatal within one year	Age- and gender-specific, adapted to Nigeria			[44,47]
**Other death**			**Distribution**	**Source #**
Probability of non-CVD mortality per year	Age- and gender-specific table in supplement			[44]
**Hypertension treatment**	**Base Case**	**Range**	**Distribution**	**Source #**
Coverage in KSHI program	29%	-	-	Kwara HH survey
SBP decrease–individuals on antihypertensive treatment (mmHg)	-20	(-31.6–-8.4)	Triangular	Kwara HH survey
SBP decrease–screened hypertensive individuals, not on antihypertensive treatment (mmHg)	-2.4	(-6.0–0)	Triangular	Kwara HH survey
**Relative risk reduction (RRR) per 10 mmHg SBP decrease**	**Base Case**	**Range**	**Distribution**	**Source #**
RRR Stroke–based on Lawes 30–44 years old	2.38	2.13–2.63	Triangular	[7]
RRR Stroke–based on Lawes 45–59 years old	2	1.92–2.04	Triangular	[7]
RRR Stroke–based on Lawes 60–69 years old	1.56	1.52–1.61	Triangular	[7]
RRR Stroke–based on Lawes 70–79 years old	1.37	1.32–1.43	Triangular	[7]
RRR CHD–based on Lawes 30–44 years old	1.92	1.54–2.38	Triangular	[7]
RRR CHD–based on Lawes 45–59 years old	1.67	1.56–1.75	Triangular	[7]
RRR CHD–based on Lawes 60–69 years old	1.33	1.27–1.39	Triangular	[7]
RRR CHD–based on Lawes 70–79 years old	1.25	1.191.32	Triangular	[7]
RRR Stroke–based on Rapsomaniki	1.16	1.14–1.18	Triangular	Calculated from[48]
RRR CHD–based on Rapsomaniki	1.16	1.15–1.18	Triangular	Calculated from[48]
**Cost parameters (2012 US$)**				
	**Base Case**	**Range**	**Distribution**	**Source #**
Cost of hypertension care per patient per year	112	101–126	Triangular	Adapted from [24]
Cost of screening per person screened	5	4–6	Triangular	[49]
Above-service delivery costs of insurance program management per patient per year	24	-	Triangular	KSHI program management
Cost of acute care after a stroke per patient	380	242–1,556	Triangular	Base Case: UITH data, [24] Range: [16,17,19,35,50–57]
Cost of follow up care after a stroke per patient per year	240	206–275	Triangular	[24]
Cost of acute care after CHD event per patient	181	115–1,180	Triangular	Base Case: UITH data, [24] Range: [16,17,19]
Cost of follow up care after CHD event per patient per year	278	235–320	Triangular	[24]
**DALY assumptions**				
	**Base Case**	**Range**	**Distribution**	
Disability weight during survival period after a fatal stroke (death during first year)	0.553	0.363–0.738	Triangular	Adapted from [27]
Disability weight during survival after a non-fatal stroke	0.256	0.021–0.553	Triangular	Adapted from [27]
Disability weight during survival period after a fatal CHD event (death during first year)	0.180	0.135–0.250	Triangular	Adapted from [27]
Disability weight during survival after a non-fatal CHD event	0.09	0.022–0.234	Triangular	Adapted from [27]
Disability weight while on antihypertensive treatment	0.031	0.017–0.05	Triangular	[27]
