# Supplementary material for: Costs and Cost-Effectiveness of Hypertension Screening and Treatment in Adults with Hypertension in Rural Nigeria in the Context of a Health Insurance Program
Source: PLoS One. 2016 Jun 27;11(6):e0157925. doi: 10.1371/journal.pone.0157925 (PMC4922631; doi:10.1371/journal.pone.0157925)
Supplement: S1 File — (DOCX) [file pone.0157925.s001.docx]

**S1 File, Supporting Information**

Cost and cost-effectiveness of hypertension screening and treatment in adults with hypertension in rural Nigeria in the context of a health insurance program: METHODS AND ADDITIONAL RESULTS

Nicole T.A. Rosendaal, Marleen E. Hendriks, et al.

June 2016

Contents

[METHODS 2](#_Toc453496476)

[A. Study setting 2](#_Toc453496477)

[B. KSHI program 2](#_Toc453496478)

[C. Population 3](#_Toc453496479)

[D. Comparators 4](#_Toc453496480)

[Intervention 4](#_Toc453496481)

[Reference scenario 5](#_Toc453496482)

[E. Health outcomes 5](#_Toc453496483)

[Fatal and non-fatal CVD events 5](#_Toc453496484)

[Non-CVD mortality 5](#_Toc453496485)

[F. Measurement of effectiveness 6](#_Toc453496486)

[Effect of intervention on systolic blood pressure 6](#_Toc453496487)

[CVD risk reduction assumptions 6](#_Toc453496488)

[G. DALYs 7](#_Toc453496489)

[Disability weights 7](#_Toc453496490)

[Survival time assumptions: fatal and non-fatal events 8](#_Toc453496491)

[H. Estimating costs 10](#_Toc453496492)

[Prevention care costs 10](#_Toc453496493)

[Acute care and follow up care costs 13](#_Toc453496494)

[ADDITIONAL RESULTS 16](#_Toc453496495)

[REFERENCES 19](#_Toc453496496)

# METHODS

## A. Study setting

The empirical data used to inform the model were derived from studies conducted in rural Kwara State. Kwara State is located in western Nigeria and is the fourth poorest state of the country.^1^ Nigeria has among the highest out-of-pocket health spending and poorest health indicators in the world.^1,2^ Similar to the rest of the country, Kwara State has a weak health system with inadequate government funding for health, weak governance and legislation, inadequate health infrastructure and poor service quality. Kwara State is participating in the federally funded National Health Insurance Scheme (NHIS). The majority of the enrollees, however, are individuals working in the formal sector. NHIS started a rural community-based social health insurance program in 2010 but access to the scheme is limited. ^1,3,4^ A baseline study conducted in 2009 in the program area, showed that less than 1% of the population was enrolled in a health insurance scheme. ^3,4^

There were few functional healthcare facilities before the start of the Kwara State Health Insurance (KSHI) program. Most clinics were primary care clinics; some provided limited secondary care (such as surgery and inpatient care). The implementing organization of the insurance program performed an assessment in the program area of potential healthcare providers that could be contracted under the insurance program. Most facilities were poorly maintained, essential equipment was lacking and patient numbers were low.

## B. KSHI program

**The Health Insurance Fund and the Kwara State Health Insurance program**

In October 2006, the Health Insurance Fund was founded, with the support of the Dutch Ministry of Foreign Affairs, to provide affordable access to quality healthcare among low-income populations in sub-Saharan Africa through the introduction of innovative financing mechanisms (including health insurance) and the improvement of healthcare quality.^5^ From the onset, the Health Insurance Fund has been working towards the following objectives:

- To increase access to quality basic health care for currently uninsured groups, mainly through private health facilities.
- To evaluate different private healthcare delivery models based on a demand-driven and results-oriented approach.
- To directly support Millennium Development Goals (MDG) 1 and 6: reducing poverty and halting the spread of HIV/AIDS, tuberculosis, malaria and other major diseases.
- To lower the threshold for investment in private healthcare infrastructure.
- To build sustainable medical and financial-administrative capacity in the health sector

To achieve these objectives, the Health Insurance Fund supports the activities of its main global implementing partner the PharmAccess Group, which employs an integrated approach of initiatives aiming to stimulate both the demand for and supply of healthcare. Interventions on the demand side include subsidized health insurance programs. On the supply side, key interventions include capacity building, quality assurance, access to credit, and involvement of the private and public sector.^5,6^ To implement these initiatives locally, the PharmAccess Group works with local partners such as private African Health Maintenance Organisations (HMOs) or health insurance companies.^3,4^

**The KSHI program in Kwara**Since 2007, Kwara State Government, Hygeia Community Health Care (local HMO), the Health Insurance Fund and PharmAccess Group have implemented the Kwara State Health Insurance (KSHI) program in rural areas of Kwara State. As of November 2015, over 120,000 people were actively enrolled and the program has been rolled out in three senatorial districts of Kwara Sate, Nigeria: Kwara North (Kwara Edu), Kwara Central (Kwara Asa), and Kwara South (Kwara Oyun).

The details and advantages of enrolment in the KSHI program were communicated by the local HMO through several activities including: face-to-face information sharing (through outreach activities to the communities, house-to-house visits by enrolment officers, health education and advocacy visits to community opinion leaders) and large-scale communication and marketing activities in the target communities (through billboards, comics, brochures, flyers and elaborate announcements and information sharing on the radio). All households living in the districts in which the program is operational are eligible for enrolment. There is no pre-enrolment screening for chronic diseases. ^3–7^

Beneficiaries are then enrolled individually (as opposed to household enrolment) on an annual basis and paid a premium of 500 NAIRA or approximately US$2.50 per person per year, at the time of the analysis (currently, the premium is 6,250 NAIRA per person per year). The premium ranges from 0.96% of the average annual per capita consumption for the lowest wealth quintile to 0.16% for the highest wealth quintile (data from baseline survey 2009). This payment represents about 8% of the total premium, the Health Insurance Fund and the Kwara State Government subsidize the remaining part of the premium. The Kwara State Government started subsidizing the premium in 2009 and its contribution has increased from 20% to 70% in 2015. The Kwara State Government has committed to take eventually over all costs of the premium subsidy. The scheme’s beneficiaries do not incur out-of-pocket costs for the services accessed within the provider network. The providers are paid directly by the insurance scheme. ^3–7^

To date, the KSHI program has contracted 38 health providers (four small health posts, 21 public facilities and 13 private facilities) to provide the care for their enrollees. Most health providers are primary and secondary care clinics with outpatient services and admission capacity. Tertiary care can be provided in two referral clinics in Ilorin (the Kwara State capital) if needed.^5–7^

**Coverage within the KSHI program in Kwara**

The insurance package covers consultations, diagnostic tests, and a selection of generic drugs for basic outpatient care, including care for hypertension and diabetes, and limited coverage of secondary care services. Secondary care services covered include radiological and more complex laboratory diagnostic tests and hospital admissions for different disease categories, minor and intermediate surgery, antenatal and delivery care, neonatal care, immunizations, and HIV screening. Excluded from the program are high technology investigations (computed tomography and magnetic resonance imaging), major surgeries and complex eye surgeries, family planning commodities, treatment for substance abuse/addiction, cancer care requiring chemotherapy and radiation therapy, provision of spectacles, contact lenses and hearing aids, dental care, intensive care treatment and dialyses. Management of acute cardiovascular events such as thrombolysis for stroke or for myocardial infarction is excluded. ^5–7^

## C. Population

The baseline survey conducted in 2009 as part of the overall evaluation of the KSHI program showed that Yoruba was the dominant ethnic group in the program area, followed by Nupe. Islam and Christianity were the main religions, trading and farming the main occupations. The baseline survey showed that 20% of the population lived below the poverty line of US $2 (PPP adjusted) per day. The population was relatively homogenous in terms of wealth with average per capita consumption ranging from 31,261 Naira (US $202) to 190,006 Naira (US $1,231) in the lowest to highest quintile.^1^ In the program area, 45.7% of the population was literate. ^3,4^ Nearly 25% of the population aged 30-79 was classified as hypertensive at the time of the baseline survey in 2009.

The distribution of relevant risk factors for cardiovascular disease (CVD) in the population was sourced from the same population-based household surveys. We characterized the individuals into 192 unique CVD risk profiles based on sex, age, blood pressure, the presence of diabetes mellitus, smoking status, and total cholesterol. Age was divided into four categories, 30-44, 45-59, 60-69 and 70-79 years, and the average age was determined per sex group for each category. Blood pressure was divided into three categories: normal blood pressure (defined as systolic blood pressure < 140 and diastolic blood pressure < 90), mild hypertension (defined as systolic blood pressure between 140-159 mmHg and/or diastolic blood pressure between 90-99 mmHg), and moderate to severe hypertension (defined as systolic blood pressure of at least 160 mmHg and/or diastolic blood pressure of at least 100 mmHg). The average blood pressure was determined for each age/sex/blood pressure sub-group.
The proportions of the population with high total cholesterol (> 5 mg/dl), smoking and diagnosed diabetes mellitus were calculated for the four age groups by sex. The observed ratios were kept constant to determine the proportions in the eight risk groups (the four male age categories and the four female age categories). The average total cholesterol and high density lipoprotein cholesterol were determined for each sex and total cholesterol group (high/low).

Since the population was assumed to have no previous CVD at the start, the proportions of left ventricle hypertrophy (LVH), atrium fibrillation (AF), and a history of CVD were kept at 0. LVH was assigned to a proportion of the population in a one-way analysis. This proportion was determined by blood pressure group and was 7.3% in the group free of hypertension, 17.9% in the group with mild hypertension and 19.2% in the group with moderate to severe hypertension. These figures were based on additional analysis on a subsample of the household survey population.

## D. Comparators

### Intervention

The intervention modelled is a population-level hypertension screening and subsequent antihypertensive treatment for high CVD risk individuals in the context of the KSHI program. We defined two strategies for treatment.

#### Risk and hypertension based strategy

In the risk and hypertension-based strategy, all individuals with hypertension stage 1 (systolic blood pressure between 140-159 mmHg and/or diastolic blood pressure between 90-99 mmHg)^8^ combined with a ten-year CVD risk greater than 20% as well as all individuals with hypertension stage 2 (systolic blood pressure of at least 160 mmHg and/or diastolic blood pressure of at least 100 mmHg )^8^, regardless of their ten-year CVD risk, were eligible for antihypertensive treatment.^9^ These groups are eligible for treatment according to the guidelines used in the KSHI program.

#### Risk based strategy

In the CVD risk based strategy, all hypertensive individuals with a ten-year CVD risk greater than 20% were eligible for treatment. This strategy is comparable to strategies tested in previous cost-effectiveness studies.^10–12^

### Reference scenario

We compared the intervention to a reference scenario in which the insurance program is not operational and people do not have access to screening or treatment for hypertension. This reference scenario was based on the observation that insurance coverage and antihypertensive treatment coverage in the hypertensive insurance target population was 0% and 4.6% respectively before the program was rolled-out. There was no information regarding the quality or consistency of the treatment received at that time.^13^ If there would have been an effect of the treatment received, this is included in the risk profiles of our population as these were based on the measured blood pressures of the population at baseline.

## E. Health outcomes

In each cycle, an individual had a probability to either stay healthy or progress to one of five end states (non-CVD mortality, fatal stroke, non-fatal stroke, fatal coronary heart disease (CHD), non-fatal CHD).

Fatal and non-fatal CVD events
We defined a fatal CVD event as a stroke or CHD event after which the patient dies within one year. A non-fatal CVD event is defined as a stroke or CHD event after which the patient survives for at least one year.

To estimate the one-year survival rate for stoke and the average duration of survival within that year (see section G, survival times), we reviewed the literature from sub-Saharan Africa (SSA). The one-year survival rate, which was used as the proportion of fatal stroke events, was 53.1% with an uncertainty range of 50 to 57.2%. We refer to section G and Table D for the full overview of data used and assumptions made.

There is a limited amount of data on survival after CHD events in SSA. Therefore, we based our figures on larger studies available conducted outside of the region. The one-year survival rate, used as the proportion of fatal CHD events, was 30.3%. We refer to section G and Table E for the full overview of data used and assumptions made.

### Non-CVD mortality

Non-CVD mortality was based on the Global Burden of Disease (GBD) study conducted in 2010.^14^ We calculated the average all-cause mortality rate for each age-category, separately by sex, and deducted the average CVD mortality rate. The same was done for the upper and lower bound of the 95% confidence interval, from which we derived the standard error (Table A).

Table A – non CVD mortality

| **Non-CVD mortality** |  |  |  |  |
| --- | --- | --- | --- | --- |
|  | **Mortality rate** | **SE** | **Distribution** | **Source:** |
| **Male:** |  |  |  |  |
| Aged 30-44 years old | 0.0063 | 0.0004 | beta | ^14^ |
| Aged 45-59 years old | 0.0085 | 0.0010 | beta | ^14^ |
| Aged 60-69 years old | 0.0169 | 0.0019 | beta | ^14^ |
| Aged 70-79 years old | 0.0385 | 0.0036 | beta | ^14^ |
| **Female:** |  |  |  |  |
| Aged 30-44 years old | 0.0064 | 0.0004 | beta | ^14^ |
| Aged 45-59 years old | 0.0077 | 0.0007 | beta | ^14^ |
| Aged 60-69 years old | 0.0180 | 0.0013 | beta | ^14^ |
| Aged 70-79 years old | 0.0370 | 0.0026 | beta | ^14^ |

Abbreviations: SE: Standard error; CVD: Cardiovascular disease.

## F. Measurement of effectiveness

Effect of intervention on systolic blood pressure
We applied blood pressure reductions for 1) individuals who were screened, told to have hypertension and given information about lifestyle measures in the household survey but who were not on pharmacological treatment during the four year follow-up survey; and 2) individuals who were treated with antihypertensive drugs. We applied blood pressure reductions as observed in the KSHI program. We previously reported on the two and four year impact of the KSHI program.^3,4^

### CVD risk reduction assumptions

Reductions in blood pressure were translated into reductions of CVD risk which were applied to the annual risks of stroke and CHD. We tested three different assumptions for risk reduction.

#### Framingham

We used the observed blood pressure reduction to recalculate the Framingham score for each individual risk profile. The Framingham risk score is meant as a clinical tool to assess an individual’s CVD risk. It is however not validated as a tool to recalculate risk within an individual after an intervention. Use of antihypertensive medication is one of the parameters in the stroke risk score for female patients.^15^ We kept this parameter constant for baseline antihypertensive treatment and thus did not incorporate it in the recalculated Framingham score.

#### Rapsomaniki

In the meta-analysis conducted by Rapsomaniki et al.^16^ relative risk reductions were calculated for 12 different presentations of cardiovascular disease. We calculated a weighted average, based on the observed distribution of coronary heart disease in a 26-year follow up of the Framingham population,^17^ of stable angina, unstable angina, myocardial infarction and unheralded CHD death to generate one relative risk reduction estimate for all CHD. We did the same for stroke, calculating a weighted average of transient ischaemic attack, ischaemic stroke, intracerebral haemorrhage, and subarachnoid haemorrhage, based on a Framingham study (for the proportion of TIAs), and two studies on stroke subtype (for ischaemic and haemorrhagic stroke) specifically in Nigeria.^15,18,19^ The 95% confidence interval around the estimates as presented in Rapsomaniki et al. were used to calculate an upper and lower bound for the stroke and CHD estimate. Table B sums up the results of these calculations.

Table B: Calculation of relative risk reduction for Rapsomaniki scenario

| **Relative risk reduction - Rapsomaniki** |  |  |  |  |
| --- | --- | --- | --- | --- |
| **Event** | Ratio  Rapsomaniki | Source | Proportion  of cases | Source |
| Stable angina | 1.19 (1.18-1.20) | ^16^ | 0.45 | ^17^ |
| Unstable angina | 1.13 (1.11-1.15) | ^16^ | 0.08 | ^17^ |
| Myocardial infarction | 1.15 (1.14-1.16) | ^16^ | 0.38 | ^17^ |
| Unheralded CHD death | 1.12 (1.10-1.15) | ^16^ | 0.09 | ^17^ |
| **Weighted average for CHD, used in study** | **1.16 (1.15-1.18)** |  |  |  |
|  |  |  |  |  |
| Transient ischaemic attack | 1.07 (1.06-1.08) | ^16^ | 0.1629 | ^15^ |
| Ischaemic stroke | 1.16 (1.14-1.18) | ^16^ | 0.4771 | ^18,19^ |
| Intracerebral haemorrhage | 1.20 (1.17-1.23) | ^16^ | 0.034 | ^18,19^ |
| Subarachnoid haemorrhage | 1.18 (1.13-1.23) | ^16^ | 0.02 | ^18,19^ |
| **Weighted average for stroke, used in study** | **1.16 (1.14-1.18)** |  |  |  |

Abbreviations: CHD: Coronary heart disease

#### Lawes

The third assumption that we tested was the relative risk reduction described by Lawes et al. ^20,21^ which was used in previous studies evaluating cost-effectiveness of CVD prevention or hypertension care in SSA.^10–12^

## G. DALYs

### Disability weights

We defined four disability weights for events (fatal strokes, non-fatal strokes, fatal CHDs and non-fatal CHDs) and one disability weight for people that are on antihypertensive treatment (Table C).

For stroke, the GBD 2010 study defined five different disability weights depending on severity; one mild, two moderate and two severe (Table C).^22^ For fatal stroke cases, we used the average of the two severe disability weights estimates for stroke as our disability weight during their survival period of less than a year. We used the highest and lowest bound of the confidence intervals of the severe stroke disability weights as the uncertainty range for the probabilistic sensitivity analysis (PSA). For non-fatal stroke cases, we used the average of the mild disability weight and the averages of the moderate and severe disability weights as the disability weight for the reminder of their life. The mild disability weight was used as a low bound and the average of the severe disability weight was used as a high bound in the PSA (Table C).

There is no overall GBD disability weight for CHD as it is a combination of several diseases. We used the proportions of myocardial infarction (MI), sudden death (SD) and angina pectoris (AP) expected in our cohort to calculate disability weights for fatal and non-fatal CHD events. The GBD study defined a mild, moderate and severe AP disability weight and two acute MI (days 1-2, days 3-28) disability weights (Table C). For fatal CHD cases, we separated CHD cases into three categories: 1) SD, 2) MI fatal within one month and 3) AP or MI fatal after surviving at least one month. Disability weights assigned to these categories were the disability weights estimates of the GBD for death (1), day 1-2 MI and severe AP respectively. To calculate a total disability weight for fatal CHD, we weighted these figures according to their relative occurrence and survival time. To calculate a range we used the upper and lower bound instead of the estimates for the same categories. For non-fatal CHD events, we used the moderate AP disability weight as the disability weight for the reminder of their life. The mild AP and the severe AP disability weight were used as the lower and upper bound respectively in the PSA (Table C). ^22^

Table C – Disability weights

| **Disability weights GBD** |  |  |  |
| --- | --- | --- | --- |
|  | **Estimate** | **Range** | **Source** |
| Acute myocardial infarction: days 1-2 | 0.422 | 0.284 – 0.556 | ^22^ |
| Acute myocardial infarction: days 3-28 | 0.056 | 0.035 – 0.082 | ^22^ |
| Angina pectoris: mild | 0.037 | 0.022 – 0.058 | ^22^ |
| Angina pectoris: moderate | 0.066 | 0.043 – 0.095 | ^22^ |
| Angina pectoris: severe | 0.167 | 0.109 – 0.234 | ^22^ |
| Heart failure: mild | 0.037 | 0.021 – 0.058 | ^22^ |
| Heart failure: moderate | 0.07 | 0.044 – 0.102 | ^22^ |
| Heart failure: severe | 0.186 | 0.128 – 0.261 | ^22^ |
| Stroke: long-term consequences, mild | 0.021 | 0.011 – 0.037 | ^22^ |
| Stroke: long-term consequences, moderate | 0.076 | 0.05 – 0.11 | ^22^ |
| Stroke: long-term consequences, moderate plus cognition problems | 0.312 | 0.211 – 0.433 | ^22^ |
| Stroke: long-term consequences, severe | 0.539 | 0.363 – 0.705 | ^22^ |
| Stroke: long-term consequences, severe plus cognition problems | 0.567 | 0.394 – 0.738 | ^22^ |
| Heart failure: mild | 0.422 | 0.284 – 0.556 | ^22^ |
| Heart failure: moderate | 0.056 | 0.035 – 0.082 | ^22^ |
| Heart failure: severe | 0.037 | 0.022 – 0.058 | ^22^ |
| Stroke: long-term consequences, mild | 0.066 | 0.043 – 0.095 | ^22^ |
| Stroke: long-term consequences, moderate | 0.167 | 0.109 – 0.234 | ^22^ |
| Generic uncomplicated disease: worry and daily medication | 0.031 | 0.017 – 0.05 | ^22^ |
|  |  |  |  |
| **Disability weights used in model** |  |  |  |
|  | **Base Case** | **Range** | **Source** |
| Disability weight during survival period after a fatal stroke (death during first year) | 0.553 | 0.363-0.738 | Weighted from ^22^ |
| Disability weight during survival after a non-fatal stroke | 0.256 | 0.021-0.553 | Weighted from ^22^ |
| Disability weight during survival period after a fatal CHD event (death during first year) | 0.180 | 0.135-0.250 | Weighted from ^22^ |
| Disability weight during survival after a non-fatal CHD event | 0.09 | 0.022-0.234 | Weighted from ^22^ |
| Disability weight while on antihypertensive treatment | 0.031 | 0.017-0.05 | ^22^ |

Abbreviations: GBD: Global burden of disease; CHD: Coronary heart disease

### Survival time assumptions: fatal and non-fatal events

To estimate the one-year survival rate for stroke and the average duration of survival within that year we reviewed the literature from SSA. All studies reporting survival rates for stroke at one week, one month, six months and/or one year were assessed. Studies were assigned a weight based on a scoring system. Each study received one base point, an additional point when they were conducted in Nigeria, an additional point when they were community-based studies (versus hospital based), and one or two additional points when the sample size was larger than 100 or 200 respectively. One-year survival estimates were used to calculate the proportion of fatal and non-fatal strokes. Estimates at one week, one month, six months and one year were used to calculate the average survival during the year after the event for fatal stroke events, taking into account the weight of the studies. The data input and weight per study is presented in Table D. The average survival for fatal stroke was estimated at 82 days. The uncertainty interval for the average time of survival during the first year was derived from the lower and upper bound of the IQR for every time period and was 78 to 90 days.

Table D – Proportion of fatal strokes and survival for fatal stroke cases

| **Stroke survival** |  |  |  |  |  |
| --- | --- | --- | --- | --- | --- |
|  | Study  Weight | **1 WEEK** | **1 MONTH*** | **6 MONTHS** | **1 YEAR** |
| **Sources:** |  | % fatal | % fatal | % fatal | % fatal |
| Alkali (2013) ^18^ | 3 | 15 | 18.8 |  |  |
| Damasenco (2010) ^23^ | 3 |  | 49.6 |  |  |
| Danesi (2013) ^24^ | 4 |  | 16.2 |  |  |
| Desalu (2011) ^25^ | 3 | 10.9 | 23.8 |  |  |
| Feigin (2009) ^26^ | 3 |  | 26.6 |  |  |
| Kengne (2006) ^27^ | 4 |  | 30 | 40 | 50 |
| Ntsehke (2013) ^28^ | 1 |  | 30 |  |  |
| Ogun (2005) ^29^ | 4 | 28 | 40 | 46 |  |
| Osuntokum (1979) ^30^ | 1 |  | 34.9 |  |  |
| Wahab (2008) ^31^ | 3 |  | 28 |  |  |
| Walker (2003) ^32^ | 2 |  | 27 | 44 |  |
| Walker (2011) ^33^ | 3 | 19 | 26.9 | 46.7 | 57.2 |

There is limited data on the long-term survival of stroke patients who survive the first year after their initial stroke. In our model, we used an estimate from a Swedish cohort that compared the expectation of remaining alive in the general population compared to stroke survivors in 1983. At the time, specialized stroke care including thrombolytic therapy with alteplase and specialized stroke units were not yet available. Therefore, these historical survival rates are more likely to reflect current survival in SSA settings compared to more recent data from high income countries. We used the observed ratio and applied it to the life expectancy in Nigeria (by age and sex) to construct an adapted life expectancy for stroke survivors in our cohort. However, standard of stroke care in Sweden in the 1980’s was probably still higher compared the standard of care in the current SSA setting. Therefore, we also halved the remaining life years after stroke in a one way sensitivity analysis.

Similarly, there is a limited amount of data on survival after CHD events in SSA. Therefore, we based our figures on larger studies available conducted outside of SSA. In order to estimate the survival after CHD, we first divided CHD events into myocardial infarction (MI), angina pectoris (AP) and sudden death (SD). We assumed the patterns observed in a 26-year follow-up of the Framingham population (Table E).^17^ The estimates for male and female individuals were used as the bounds for the uncertainty interval. We then calculated an average survival and average fatal proportion separately for MI, AP and SD. These figures were then summarized into one overarching figure for the proportion of fatal CHD events and for the mean survival of the fatal CHD events. The calculation of the lower and upper bound were constructed in the same manner (Table E).

Table E – CHD, breakdown of events, one year case fatality and mean survival one year fatal state

| **Coronary Heart Disease - Survival** |  |  |  |  |
| --- | --- | --- | --- | --- |
|  |  |  |  | Source: |
| Breakdown of CHD events | Myocardial  Infarction | Angina  Pectoris | Sudden  Death |  |
| Mean (%) | 38 | 53 | 9 | ^17^ |
| Upper bound (%) | 43 | 47 | 10 | ^17^ |
| Lower bound (%) | 29 | 64 | 7 | ^17^ |
|  |  |  |  |  |
| Case fatality in Myocardial Infarction - Calculation | 1 Hour | 1 Month | 1 Year |  |
| Case fatality (%) | 23 | 36 | 42 | ^34^ |
|  |  |  |  |  |
| Case fatality in Sudden Death | 100% case fatality | | |  |
|  |  |  |  | Source: |
| Case fatality in Angina Pectoris | 10% in year 1 | | | ^34–36^ |
|  |  |  |  |  |
|  | Myocardial  Infarction | Angina  Pectoris | Sudden  Death | Total |
| Combined total fatal CHD rate (% of CHD * % fatal) | 16.0% | 5.3% | 9.0% | **30.26%** |
|  |  |  |  |  |
| Average survival for 1-year fatal CHD |  |  |  | **49.30 days** |
| Summary: | Base case | Upper *  bound | Lower *  bound |  |
| Case fatality (%) | 30.26 | 25.58 | 32.76 |  |
| Average survival (days) | 49.30 | 44.3 | 61.31 |  |

Abbreviations: CHD: Coronary heart disease

To estimate the expected survival time for CHD survivors (non-fatal CHD), cohort data for AP and MI from a 15-year study of CHD survivors was used.^35^ Comparing the survival likelihood after year one with WHO mortality tables, we found an increase in the probability of dying within 14 years after an AP or MI event as compared with non-CVD mortality. We then calculated the relative increase in mortality for both AP and MI and weighted according to their relative occurrence for a total rate increase for CHD.

## H. Estimating costs

### Prevention care costs

The costs of delivering antihypertensive treatment included population-based screening costs, service costs for antihypertensive treatment and above-service delivery costs associated with the local operations of the insurer and program management.

#### Screening costs

The costs of population-based screening from a healthcare provider perspective was derived from WHO estimates.^37^ The cost of screening per person screened was assigned to all individuals in the cohort.

#### Service costs

The service delivery cost from a healthcare provider perspective for hypertension was estimated from a costing study undertaken in the Ogo Oluwa Hospital, a private hospital participating in the Kwara State Health Insurance program.^38^ A comprehensive overview of the methods used in this costing study can be found in the original publication.^38^ Table F provides an overview of the included costs. The cost in the current study differ slightly from the cost of US$ 118 for hypertension care indicated in the costing study. The costing study included statins for a portion of the hypertensive patients. As we observed no statin use in the impact study, costs of statins were excluded from our final estimate resulting in a slightly lower price for antihypertensive treatment care. The costing study included a sensitivity analysis in which different parameters were varied. The parameters with the largest impact were a change in productive work hours and a change in drug prices. Variation in these parameters were used to create the lower and upper bound of the total costs. More details can be found in the costing study. ^38^

Table F – Costs antihypertensive treatment care ^38^

| **Cost parameters** |  |  |  |
| --- | --- | --- | --- |
|  | **Unit Costs**  **(2012 US$)** | **Utilization per year** | **Yearly costs**  **(2012 US$)** |
| **Training** | 0.33 | 1 | **0.33** |
| **Consultations** | 0.53 | 12 | **6.37** |
| **Tests** |  |  |  |
| Quality control Reflotron | 3.60 | 1 | 3.60 |
| Reflotron Glucose | 3.12 | 1 | 3.12 |
| Reflotron Total Cholesterol | 3.61 | 1 | 3.61 |
| Reflotron Triglycerides | 3.61 | 1 | 3.61 |
| **Reflotron Potassium** | 3.79 | 1 | 3.79 |
| Reflotron HDL Cholesterol | 3.63 | 1 | 3.63 |
| Reflotron Creatinine | 3.67 | 1 | 3.67 |
| Microalbuminuria | 2.97 | 1 | 2.97 |
| Draw blood | 0.15 | 1 | 0.15 |
| ECG test | 3.17 | 1 | 3.17 |
| **Sub-total (tests)** |  |  | **31.31** |
| **Drugs:** |  |  |  |
| Nifedipine SR 20mg | 0.01490 | 2.2 * 365.25 | 11.97 |
| Captopril 25mg | 0.02267 | 0.9 * 365.25 | 7.45 |
| Moduretic 25/5mg | 0.02267 | 0.5 * 365.25 | 4.14 |
| Vasoprin 75mg | 0.00972 | 0.1 * 365.25 | 0.35 |
| Methyldopa 250mg | 0.03563 | 0.1 * 365.25 | 1.30 |
| **Sub-total (drugs)** |  |  | **25.22** |
| **Total Direct Costs** |  |  | **63.23** |
| Mark-up for indirect costs (76.42%) |  |  | **48.32** |
| **Total antihypertensive treatment care cost:** |  |  | **111.54 (100.98-125.85)** |

Abbreviations: HDL: high density lipoprotein; ECG: electrocardiogram

The costing study also included alternative scenarios. A combination of task-shifting from doctors to nurses, minimal target organ damage screening and a reduction in the number of consultations per year led to the largest reduction in cost.^38^ We used these reduced costs in a one-way sensitivity analysis (Table G).

Table G – Costs antihypertensive treatment care – reduced cost scenario

| **Cost parameters** |  |  |  |
| --- | --- | --- | --- |
|  | **Unit Costs**  **(2012 US$)** | **Utilization** | **Yearly costs**  **(2012 US$)** |
| **Training** | 0.33 | 1 | **0.33** |
| **Consultations** | 0.39 | 6 | **2.36** |
| **Tests** |  |  |  |
| Urine dipstick (albuminuria) | 0.37 | 1 | 0.37 |
| Quality control Accutrend | 0.82 | 1 | 0.82 |
| Accutrend Plus Glucose | 0.95 | 1 | 0.95 |
| Accutrend Plus Cholesterol | 2.04 | 1 | 2.04 |
| Draw blood | 0.15 | 1 | 0.15 |
| **Sub-total (tests)** |  |  | **4.33** |
| **Drugs:** |  |  |  |
| Nifedipine SR 20mg | 0.01490 | 2.2 * 365.25 | 11.97 |
| Captopril 25mg | 0.02267 | 0.9 * 365.25 | 7.45 |
| Moduretic 25/5mg | 0.02267 | 0.5 * 365.25 | 4.14 |
| Vasoprin 75mg | 0.00972 | 0.1 * 365.25 | 0.35 |
| Methyldopa 250mg | 0.03563 | 0.1 * 365.25 | 1.30 |
| **Sub-total (drugs)** |  |  | **25.22** |
| **Total Direct Costs** |  |  | **32.24** |
| Mark-up for indirect costs (76.42%) |  |  | **24.63** |
| **Total antihypertensive treatment care cost:** |  |  | **56.87 (46.31-67.43)** |

#### Above-service delivery costs

The section below is based on Gomez et al.^39^ This study assessed the cost and cost-effectiveness of maternal care provided within the KSHI program.

Above-service delivery costs associated with the local operations of the HMO (HCHC) and program management at PharmAccess level were also included. In determining the cost-effectiveness of the program, these costs were taken into account from the beginning of the program in 2006 until 2018. After this date the program is expected to be transferred to the Kwara state Government. Expenses over the period 2006-2013 are audited, while from 2014 the amounts are based on projections.

In order to account for all costs related to the operational activities of the insurer and program management at PharmAccess level, we divided the ‘above-service’ delivery costs into three components:

1. Costs related to operational activities at HMO Hygeia Community Health Care (HCHC)

level, including costs of scaling up the program;

2. Costs related to the upgrading of the health care providers in the network;

3. Costs related to technical assistance delivered by PharmAccess Foundation.

The first component covers the operational costs at HCHC level. This includes costs of marketing activities and administration of the insurance product. For these costs, HCHC receives an income from the insurance premium paid by enrollees. Since sufficient scale has not been achieved, additional investment from the HIF is required to cover all expenditures. Once scale has been achieved, it is expected that HCHC will no longer receive the additional income. In this analysis, we accounted for the additional investment required up to 2018 when it is expected that the program will have achieved the necessary scale and become sustainable. Over the period 2007-2014, HCHC was involved in the operational activities of three insurance programs in Nigeria (one rural – the KSHI, and two urban), we allocated HCHC’s overheads over the three programs according to volume of enrollees during this period. In 2014, the urban programs were terminated and from that moment onwards, HCHC’s overheads had been allocated to the KSHI program exclusively.

The second component refers to expenses upgrading facilities entering the KSHI program. All clinics in the program have to meet a certain minimum criteria of quality of care. Therefore, in most cases clinics require upgrading before entering the program.

The last component refers to technical assistance delivered by PharmAccess Foundation. Technical assistance consists of: health plan design, data-management, participation in the SafeCare program and additional quality improvement trainings. In particular, all clinics in KSHI programs are enrolled in the SafeCare program, in which they are assessed and receive quality improvement reports. In this analysis, expenses related to operational research and impact evaluation were not taken into account.

We accounted for start-up costs of the program in 2006 (enrolment began in 2007). A contract with Kwara State Government has been signed to take over the responsibility of the program at the end of 2018. Therefore, the above-service delivery costs have been calculated over the 2006-2018 period. For the years 2006 to 2013, financial records audited by accountants and consistent with subsidy statements from donors were used. The amounts related to 2014 to 2016 are based on budgets. From 2016 to 2018, the figures are based on projections. Although the Kwara State Governor signed a contract to enroll 600.000 people in the program by 2018, a lower target has been assumed in this analysis (a conservative assumption). As of December 2014, the program had reached 40% of its enrolment target. Therefore, as a conservative assumption, it is assumed that 40% of the original target will be reached by 2018 (40%*600,000 = 243,000 people enrolled in December 2018), which represents the basis for our projections.

The weighted average (weighted by number of enrollees per year) of above-service delivery costs per enrollee per year, over the full program period, was estimated at 2012 US$ 24.10 (Table H). We added this as a mark-up to all patients on antihypertensive treatment in in the intervention scenario.

Table H – Overview of the above-service delivery costs for the period 2006-2018.

| **Cost parameters** |  |  |  |
| --- | --- | --- | --- |
|  | **2006-2013**  **(realized costs)** | **2014-2018**  **(projected costs)** | **2006-2018**  **( costs yearly, 2012 US$)** |
| Operational costs | 10.91 | 9.98 | **10.24** |
| Upgrading costs | 1.05 | 0.76 | **0.84** |
| Technical assistance costs | 22.63 | 9.38 | 13.02 |
| **Total** | 34.60 | 20.13 | 24.10 |

### Acute care and follow up care costs

Acute care costs included costs related to care directly after the event. Costs included in-hospital stay, tests, and drugs. These were estimated using an ingredients approach. We combined data from the costing study ^38^ with additional data collected from the University of Ilorin Teaching Hospital (UITH) for the current study. The number of in-hospital days were based on data of 186 stroke cases collected at UITH. The data collected at UITH originated from a register of all acute stroke cases seen and additionally from a more extensive questionnaire completed by the doctors for patients with acute stroke. Both data sources contained information on the length of stay. We assumed the same utilization for CHD. While different CHDs may require more or less hospitalization days, milder cases may present to the hospital and need tests more than once. Since we do not model (costs of) subsequent events, we assumed a one-time acute care cost for all cases. The utilization of tests and drugs was based on a standard set of basic tests that would be required for a CVD event (separate for stroke and CHD). A detailed overview of included costs is given in Table I. We used a wide uncertainty range in the PSA. The ranges for stroke care were constructed from literature, excluding studies from South Africa and excluding thrombolysis and costs based on care in a specialized stroke unit.^10,12,27,40–48^ For care after a CHD event, there were few estimates in the literature, and our estimate was the lowest estimate. We used the highest estimate from literature and constructed a lower bound for CHD, using the relative difference between the lowest bound and our point estimate for stroke.

Table I - Costs CVD event – acute

| **Cost parameters** |  |  |  |  |
| --- | --- | --- | --- | --- |
|  | **Unit Costs**  **(2012 US$)** | **Utilization** | **Total Costs**  **(2012 US$)** | **Source** |
| **Acute care costs stroke event per patient** |  |  |  |  |
| **Tests:** |  |  |  |  |
| Tests prevention care costs  (as in Table F, including indirect costs) | 55.25 | 1 | 55.25 | ^38^ |
| CT scan | 185.11 | 1 | 185.11 | UITH – own calculation |
| Carotid dopler scan | 15.43 | *0.64 # | 9.87 | UITH – own calculation |
| Kidney: Na | 2.16 | 1 | 2.16 | UITH – own calculation |
| kidney: Urea | 2.16 | 1 | 2.16 | UITH – own calculation |
| **Total tests** |  |  | **254.55** |  |
| **Drugs:** |  |  |  |  |
| Nifedipine SR 20mg | 0.01490 | 2.2 * 13 days | 0.43 | ^38^ |
| Captopril 25mg | 0.02267 | 0.9 * 13 days | 0.27 | ^38^ |
| Moduretic 25/5mg | 0.02267 | 0.5 * 13 days | 0.15 | ^38^ |
| Vasoprin 75mg | 0.00972 | 0.64*13 days | 0.08 | ^38^ |
| Methyldopa 250mg | 0.03563 | 0.1 * 13 days | 0.05 | ^38^ |
| IV fluids 24 hour (1.5 liter) | 1.85 | 1 day | 1.85 | UITH – own calculation |
| Simvastatine 20 mg | 0.09716 | 2*13 days | 2.53 | ^38^ |
| **Total drugs**  **(including indirect costs for costing study figures)** |  |  | **8.01** |  |
| **Hospital bed days:** |  |  |  |  |
| Emergency ward | 7.40 | 1 day | **7.40** | UITH – own calculation |
| Normal ward – costs first 5 days US$ 61.7  after that a charge of US$ 1.23 per day | 61.7  1.23 | 1 time  7 days | **70.34** | UITH – own calculation |
| **Additional costs:** |  |  |  |  |
| Physiotherapy / speech / occupational therapy | 3.09 | 13 days | **40.11** | UITH – own calculation |
| **Total cost of acute care after a stroke per patient** |  |  | **380.42** |  |
|  |  |  |  |  |
| **Acute care costs CHD event per patient** |  |  |  |  |
| **Tests:** |  |  |  |  |
| Tests prevention care costs  (as in Table F, including indirect costs) | 55.25 | 1 | 55.25 | ^38^ |
| Echocardiogram | 30.85 | 1 | 30.85 | UITH – own calculation |
| Kidney: Na | 2.16 | 1 | 2.16 | UITH – own calculation |
| kidney: Urea | 2.16 | 1 | 2.16 | UITH – own calculation |
| **Total tests** |  |  | **90.42** |  |
| **Drugs:** |  |  |  |  |
| Nifedipine SR 20mg | 0.01490 | 2.2 * 13 days | 0.43 | ^38^ |
| Captopril 25mg | 0.02267 | 0.9 * 13 days | 0.27 | ^38^ |
| Moduretic 25/5mg | 0.02267 | 0.5 * 13 days | 0.15 | ^38^ |
| Vasoprin 75mg | 0.00972 | 1*13 days | 0.13 | ^38^ |
| Methyldopa 250mg | 0.03563 | 0.1 * 13 days | 0.05 | ^38^ |
| IV fluids 24 hour (1.5 liter) | 1.85 | 1 day | 1.85 | UITH – own calculation |
| Simvastatine 20 mg | 0.09716 | 2*13 days | 2.53 | ^38^ |
| Propanalol (80 mg) | 0.02731 | 2*13 days | 0.71 | ^38^ |
| **Total drugs**  **(including indirect costs for costing study figures)** |  |  | **9.34** |  |
| **Hospital bed days:** |  |  |  |  |
| Emergency ward | 7.40 | 1 day | **7.40** | UITH – own calculation |
| Normal ward – costs first 5 days US$ 61.7  after that a charge of US$ 1.23 per day | 61.7  1.23 | 1 time  7 days | **70.34** | UITH – own calculation |
| **Additional costs:** |  |  |  |  |
| Physiotherapy | 3.09 | 1 days | **3.09** | UITH – own calculation |
| **Total cost of acute care CHD per patient** |  |  | **180.59** |  |

#, only for ischaemic strokes, 64% of cases, based on two recent studies in Nigeria which used MRI/CT scan to determine the pathological type of stroke. Weighted average based on sample size: 36% hemorrhagic, 64% ischaemic. ^18,19^
Abbreviations: CT: Computerized tomography; UITH: University of Illorin Teaching Hospital; CHD: Coronary heart disease;

Follow-up care costs were assumed to be equal to antihypertensive treatment care costs with the addition of a number of drugs, depending on the event. Costs were again sourced from the costing study undertaken in the Ogo Oluwa Hospital.^38^ Table J provides an overview of the included costs. For the one-way analysis with the reduced costs, the costs of follow-up care decreased as well and were US$ 185.5 for stroke and US$ 222.8 for CHD.

Table J – Follow-up costs CVD events ^38^

| **Follow-up care costs stroke event** |  |  |  |
| --- | --- | --- | --- |
|  | **Unit Costs (2012 US$)** | **Utilization** | **Total Costs (2012 US$)** |
| **Training** | 0.33 | 1 | **0.33** |
| **Consultations** | 0.53 | 12 | **6.37** |
| **Tests** |  |  |  |
| Quality control Reflotron | 3.60 | 1 | 3.60 |
| Reflotron Glucose | 3.12 | 1 | 3.12 |
| Reflotron Total Cholesterol | 3.61 | 1 | 3.61 |
| Reflotron Triglycerides | 3.61 | 1 | 3.61 |
| **Reflotron Potassium** | 3.79 | 1 | 3.79 |
| Reflotron HDL Cholesterol | 3.63 | 1 | 3.63 |
| Reflotron Creatinine | 3.67 | 1 | 3.67 |
| Microalbuminuria | 2.97 | 1 | 2.97 |
| Draw blood | 0.15 | 1 | 0.15 |
| Electrocardiogram | 3.17 | 1 | 3.17 |
| **Total tests** |  |  | **31.31** |
| **Drugs** |  |  |  |
| Nifedipine SR 20mg | 0.01490 | 2.2 * 365.25 | 11.97 |
| Captopril 25mg | 0.02267 | 0.9 * 365.25 | 7.45 |
| Moduretic 25/5mg | 0.02267 | 0.5 * 365.25 | 4.14 |
| Vasoprin 75mg | 0.00972 | 0.64*365.25 | 2.27 |
| Methyldopa 250mg | 0.03563 | 0.1 * 365.25 | 1.30 |
| Simvastatine 20 mg | 0.09716 | 2 * 365.25 | 70.97 |
| **Total drugs** |  |  | **98.11** |
| **Total direct costs** |  |  | **136.12** |
| **Total costs (including 76.42% indirect)** |  |  | **240.14 (205.52-274.76)** |
| **Follow-up care costs CHD event** |  |  |  |
| **Training** | 0.33 | 1 | **0.33** |
| **Consultations** | 0.53 | 12 | **6.37** |
| **Tests** |  |  |  |
| Quality control Reflotron | 3.60 | 1 | 3.60 |
| Reflotron Glucose | 3.12 | 1 | 3.12 |
| Reflotron Total Cholesterol | 3.61 | 1 | 3.61 |
| Reflotron Triglycerides | 3.61 | 1 | 3.61 |
| **Reflotron Potassium** | 3.79 | 1 | 3.79 |
| Reflotron HDL Cholesterol | 3.63 | 1 | 3.63 |
| Reflotron Creatinine | 3.67 | 1 | 3.67 |
| Microalbuminuria | 2.97 | 1 | 2.97 |
| Draw blood | 0.15 | 1 | 0.15 |
| Electrocardiogram | 3.17 | 1 | 3.17 |
| **Total tests** |  |  | **31.31** |
| **Drugs** |  |  |  |
| Nifedipine SR 20mg | 0.01490 | 2.2 * 365.25 | 11.97 |
| Captopril 25mg | 0.02267 | 0.9 * 365.25 | 7.45 |
| Moduretic 25/5mg | 0.02267 | 0.5 * 365.25 | 4.14 |
| Vasoprin 75mg | 0.00972 | 1 * 365.25 | 3.55 |
| Methyldopa 250mg | 0.03563 | 0.1 * 365.25 | 1.30 |
| Simvastatine 20 mg | 0.09716 | 2 * 365.25 | 70.97 |
| Propanolol 80 mg | 0.02731 | 2 * 365.25 | 19.87 |
| **Total drugs** |  |  | **119.27** |
| **Total direct costs** |  |  | **157.27** |
| **Total costs (including 76.42% indirect)** |  |  | **277.46 (235.37-319.54)** |

Abbreviations: HDL: High density lipoprotein

# ADDITIONAL RESULTS

Fig A: CE plane


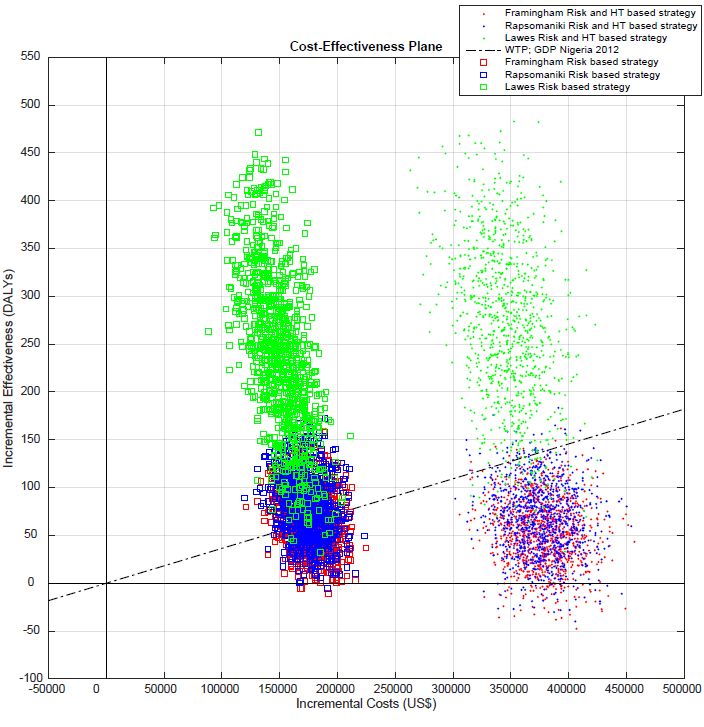


GDP Nigeria 2012: US$ 2,742; Framingham: assuming recalculation of Framingham equation; Rapsomaniki: assuming relative risk reduction based on Rapsomaniki^16^; Lawes: assuming relative risk reduction based on Lawes^20^

Table K; one-way analysis Risk and HT based strategy

| **Parameter** |  | **Assumption** | **ICER (mean) Framingham** | **ICER (mean) Rapsomaniki** | **ICER (mean) Lawes** |
| --- | --- | --- | --- | --- | --- |
| **Discounting** | Base case | 0.03 | 7,815 | 6,256 | 1,406 |
|  | High | 0.10 | 672,459 | 53,567 | 3,370 |
|  | Low | 0.00 | 4,387 | 3,706 | 956 |
| **Above-service delivery costs** | Base case | 24.10 | 7,815 | 6,256 | 1,406 |
|  | High | 120.5 | 12,921 | 10,355 | 2,388 |
|  | Low | 12.05 | 7,177 | 5,743 | 1,283 |
| **Disability weight for hypertension treatment** | Base case | 0.031 | 7,815 | 6,256 | 1,406 |
|  | alternative | 0 | 2,989 | 2,724 | 1,072 |
| **Cost prevention care*** | Base case | 111.54 | 7,815 | 6,256 | 1,406 |
|  | Alternative | 56.87 | 4,969 | 3,974 | 873 |
| **Comparable to other studies (coverage, effect and disability weight for treatment)** | Base case | 29%,  20 mmHg, 0.031 | 7,815 | 6,256 | 1,406 |
|  | Alternative | 100%,  14.6 mmHg, 0 | 5,395 | 4,856 | 2,029 |
| **Effectiveness of treatment** | Base case |  | 7,815 | 6,256 | 1,406 |
|  | High | -31.6 | 4,260 | 3,717 | 1,139 |
|  | Low | -8.4 | 80,037 | 29,306 | 2,194 |
| **Proportion of fatal Stroke events** | Base case | 0.3026 | 7,815 | 6,256 | 1,406 |
|  | High | 0.45 | 6,779 | 5,657 | 1,328 |
|  | Low | 0.15 | 9,754 | 7,252 | 1,523 |
| **Survival non-fatal events** | Base case | Age-gender specific | 7,815 | 6,256 | 1,406 |
|  | Alternative | Halved | 5,812 | 4,812 | 1,236 |
| **Proportion of fatal CHD events** | Base case | 0.5309 | 7,815 | 6,256 | 1,406 |
|  | High | 0.75 | 7,221 | 5,774 | 1,356 |
|  | Low | 0.25 | 8,548 | 6,853 | 1,461 |
| **Acute cost stroke** | Base case | 380.4 | 7,815 | 6,256 | 1,406 |
|  | High | 1,556.4 | 7,630 | 6,127 | 1,327 |
|  | Low | 241.94 | 7,837 | 6,271 | 1,415 |
| **Acute cost CHD event** | Base case | 180.60 | 7,815 | 6,256 | 1,406 |
|  | High | 1,180.1 | 7,657 | 6,083 | 1,325 |
|  | Low | 114.9 | 7,826 | 6,267 | 1,411 |
| **Prevalence of LVH** | Base case | 0 | 7,815 | 6,256 | 1,406 |
|  | Alternative | HT 0: 0.073  HT 1: 0.179  HT 2: 0.192 | 6,096 | 5,164 | 1,234 |

* Costs of secondary prevention care (follow-up care) in the alternative model: US$ 185.46 for stroke, US$ 222.78 for CHD. Rapsomaniki: assuming relative risk reduction based on Rapsomaniki^16^; Lawes: assuming relative risk reduction based on Lawes^20^ Abbreviations: ICER: Incremental cost effectiveness ratio; CHD: Coronary heart disease

Table L; one-way analysis Risk based strategy

| **Parameter** |  | **Assumption** | **ICER (mean) Framingham** | **ICER (mean) Rapsomaniki** | **ICER (mean) Lawes** |
| --- | --- | --- | --- | --- | --- |
| **Discounting** | Base case | 0.03 | 2,959 | 2,498 | 732 |
|  | High | 0.10 | 7,433 | 5,956 | 1,570 |
|  | Low | 0.00 | 2,023 | 1,734 | 509 |
| **Above-service delivery costs** | Base case | 24.10 | 2,959 | 2,498 | 732 |
|  | High | 120.5 | 4,653 | 3,937 | 1,202 |
|  | Low | 12.05 | 2,747 | 2,318 | 673 |
| **Disability weight for hypertension treatment** | Base case | 0.031 | 2,959 | 2,498 | 732 |
|  | alternative | 0 | 1,926 | 1,716 | 637 |
| **Cost prevention care*** | Base case | 111.54 | 2,959 | 2,498 | 732 |
|  | Alternative | 56.87 | 2,026 | 1,708 | 487 |
| **Comparable to other studies (coverage, effect and no disability weight for treatment)** | Base case | 29%,  20 mmHg, 0.031 | 2,959 | 2,498 | 732 |
|  | Alternative | 100%,  14.6 mmHg, 0 | 3,691 | 3,211 | 1,372 |
| **Effectiveness of treatment** | Base case |  | 2,959 | 2,498 | 732 |
|  | High | -31.6 | 2,192 | 1,895 | 633 |
|  | Low | -8.4 | 4,718 | 3,930 | 933 |
| **Proportion of fatal Stroke events** | Base case | 0.3026 | 2,959 | 2,498 | 732 |
|  | High | 0.45 | 2,729 | 2,352 | 705 |
|  | Low | 0.15 | 3,324 | 2,717 | 772 |
| **Survival non-fatal events** | Base case | Age-gender specific | 2,959 | 2,498 | 732 |
|  | Alternative | Halved | 2,496 | 2,131 | 675 |
| **Proportion of fatal CHD events** | Base case | 0.5309 | 2,959 | 2,498 | 732 |
|  | High | 0.75 | 2,834 | 2,383 | 715 |
|  | Low | 0.25 | 3,101 | 2,630 | 750 |
| **Acute cost stroke** | Base case | 380.4 | 2,959 | 2,498 | 732 |
|  | High | 1,556.4 | 2,840 | 2,409 | 657 |
|  | Low | 241.94 | 2,973 | 2,508 | 740 |
| **Acute cost CHD event** | Base case | 180.60 | 2,959 | 2,498 | 732 |
|  | High | 1,180.1 | 2,861 | 2,382 | 659 |
|  | Low | 114.9 | 2,965 | 2,505 | 736 |
| **Prevalence of LVH** | Base case | 0 | 2,959 | 2,498 | 732 |
|  | Alternative | HT 0: 0.073  HT 1: 0.179  HT 2: 0.192 | 2,624 | 2,277 | 669 |

* Costs of secondary prevention care (follow-up care) in the alternative model: US$ 185.46 for stroke, US$ 222.78 for CHD. Rapsomaniki: assuming relative risk reduction based on Rapsomaniki^16^; Lawes: assuming relative risk reduction based on Lawes^20^ Abbreviations: ICER: Incremental cost effectiveness ratio; CHD: Coronary heart disease

# REFERENCES

1. Gustafsson-Wright E, Schellekens O. Achieving Universal Health Coverage in Nigeria One State at a Time: A Public-Private Partnership Community-Based Health Insurance Model. Washington, DC; 2013. http://www.brookings.edu/~/media/research/files/papers/2013/06/achieving%20universal%20health%20coverage%20nigeria%20gustafsson%20wright/achieving%20universal%20health%20coverage%20in%20nigeria.pdf. Accessed October 8, 2014.

2. World Bank. World Development Indicators. http://data.worldbank.org/indicator. Published 2012. Accessed October 8, 2014.

3. Hendriks ME, Wit FWNM, Akande TM, et al. Effect of Health Insurance and Facility Quality Improvement on Blood Pressure in Adults With Hypertension in Nigeria: A Population-Based Study. JAMA Intern Med. 2014;174(4):555-563. doi:10.1001/jamainternmed.2013.14458.

4. Hendriks ME, Rosendaal NTA, Wit FWNM, et al. Sustained effect of health insurance and facility quality improvement on blood pressure in adults with hypertension in Nigeria: A population-based study. Int J Cardiol. 2016;202:477-484. doi:10.1016/j.ijcard.2015.09.036.

5. Health Insurance Fund. Health Insurance Fund Website. http://hifund.org/. Published 2014. Accessed October 8, 2014.

6. Pharmaccess Foundation. PharmAccess Foundation website. http://pharmaccess.org/RunScript.asp?p=ASP\Pg0.asp. Accessed March 13, 2015.

7. Hygeia group. Hygeia Community Health Care. http://www.hygeiagroup.com/Businesses/HCHC.aspx. Accessed August 10, 2015.

8. National High Blood Pressure Education Program. The seventh report of the Joint National Committee on prevention, detection, evaluation, and treatment of high blood pressure. 2004. http://www.ncbi.nlm.nih.gov/books/NBK9621/. Accessed November 28, 2014.

9. World Health Organization, UNAIDS. Prevention of Cardiovascular Disease. World Health Organization; 2007. https://books.google.com/books?hl=en&lr=&id=AS2RmtQVuLwC&oi=fnd&pg=PT5&dq=%22frontiers+or+boundaries.+Dotted+lines+on+maps+represent+approximate+border+lines+for+which%22+%22contained+in+this+publication.+However,+the+published+material+is+being%22+%22and+cerebrovascular+events+(strokes)+frequently+occur+suddenly,%22+&ots=AILod4_6LK&sig=iquvRZ98OzZ1Oc3wAofESRM-GPM. Accessed August 13, 2015.

10. Gaziano TA, Opie LH, Weinstein MC. Cardiovascular disease prevention with a multidrug regimen in the developing world: a cost-effectiveness analysis. The Lancet. 2006;368(9536):679–686.

11. Murray CJ, Lauer JA, Hutubessy RC, et al. Effectiveness and costs of interventions to lower systolic blood pressure and cholesterol: a global and regional analysis on reduction of cardiovascular-disease risk. The Lancet. 2003;361(9359):717–725.

12. Ortegon M, Lim S, Chisholm D, Mendis S. Cost effectiveness of strategies to combat cardiovascular disease, diabetes, and tobacco use in sub-Saharan Africa and South East Asia: mathematical modelling study. BMJ. 2012;344:e607-e607. doi:10.1136/bmj.e607.

13. Hendriks ME, Wit FWNM, Akande TM, et al. Effect of Health Insurance and Facility Quality Improvement on Blood Pressure in Adults With Hypertension in Nigeria: A Population-Based Study. JAMA Intern Med. 2014;174(4):555. doi:10.1001/jamainternmed.2013.14458.

14. Institute for Health Metrics and Evaluation (IHME). Nigeria Global Burden of Disease Study 2010 (GBD 2010) Results 1990-2010. http://ghdx.healthdata.org/record/nigeria-global-burden-disease-study-2010-gbd-2010-results-1990-2010. Published 2014. Accessed September 29, 2014.

15. Wolf PA, D’Agostino RB, Belanger AJ, Kannel WB. Probability of stroke: a risk profile from the Framingham Study. Stroke. 1991;22(3):312-318. doi:10.1161/01.STR.22.3.312.

16. Rapsomaniki E, Timmis A, George J, et al. Blood pressure and incidence of twelve cardiovascular diseases: lifetime risks, healthy life-years lost, and age-specific associations in 1· 25 million people. The Lancet. 2014;383(9932):1899–1911.

17. Lerner DJ, Kannel WB. Patterns of coronary heart disease morbidity and mortality in the sexes: A 26-year follow-up of the Framingham population. Am Heart J. 1986;(111):383-390.

18. Alkali NH, Bwala SA, Akano AO, Osi-Ogbu O, Alabi P, Ayeni OA. Stroke risk factors, subtypes and 30-day case fatality in Abuja, Nigeria. Niger Med J. 2013;54(2):129-135.

19. Yunusha GH, Saidu SA, Ma’aji SM, Danfulani M. Pattern of computerized tomography of the brain findings in stroke patients in Sokoto, northwestern Nigeria. Ann Afr Med. 2014;13(4):217-220.

20. Lawes CM, Vander Hoorn S, Law MR, Elliott P, MacMahon S, Rodgers A. High blood pressure. In: Comparative Quantification of Health Risks: Global and Regional Burden of Disease Attributable to Selected Major Risk Factors. Geneva: World Health Organization; 2004:281–390. http://apps.who.int/iris/bitstream/10665/42792/1/9241580348_eng_Volume1.pdf#page=305. Accessed September 19, 2014.

21. Asia Pacific Cohort Studies Collaboration. Blood Pressure Indices and Cardiovascular Disease in the Asia Pacific Region: A Pooled Analysis. Hypertension. 2003;42(1):69-75. doi:10.1161/01.HYP.0000075083.04415.4B.

22. Salomon JA, Vos T, Hogan DR, et al. Common values in assessing health outcomes from disease and injury: disability weights measurement study for the Global Burden of Disease Study 2010. The Lancet. 2013;380(9859):2129–2143.

23. Damasceno A, Gomes J, Azevedo A, et al. An Epidemiological Study of Stroke Hospitalizations in Maputo, Mozambique: A High Burden of Disease in a Resource-Poor Country. Stroke. 2010;41(11):2463-2469. doi:10.1161/STROKEAHA.110.594275.

24. Danesi MA, Okubadejo NU, Ojini FI, Ojo OO. Incidence and 30-day case fatality rate of first-ever stroke in urban Nigeria: The prospective community based Epidemiology of Stroke in Lagos (EPISIL) phase II results. J Neurol Sci. 2013;331(1-2):43-47. doi:10.1016/j.jns.2013.04.026.

25. Desalu OO, Wahab KW, Fawale B, et al. A review of stroke admissions at a tertiary hospital in rural Southwestern Nigeria. Ann Afr Med. 2011;10(2):80-85.

26. Feigin VL, Lawes CM, Bennett DA, Barker-Collo SL, Parag V. Worldwide stroke incidence and early case fatality reported in 56 population-based studies: a systematic review. Lancet Neurol. 2009;8(4):355–369.

27. Kengne AP, Anderson CS. The neglected burden of stroke in Sub-Saharan Africa. Int J Stroke. 2006;1(4):180–190.

28. Ntsekhe M, Damasceno A. Recent advances in the epidemiology, outcome, and prevention of myocardial infarction and stroke in sub-Saharan Africa. Heart. 2013;99(17):1230-1235. doi:10.1136/heartjnl-2012-303585.

29. Ogun SA, Ojini FI, Ogungbo B, Kolapo KO, Danesi MA. Stroke in South West Nigeria: A 10-Year Review. Stroke. 2005;36(6):1120-1122. doi:10.1161/01.STR.0000166182.50840.31.

30. Osuntokun BO, Bademosi O, Akinkugbe OO, Oyediran AB, Carlisle R. Incidence of stroke in an African City: results from the Stroke Registry at Ibadan, Nigeria, 1973-1975. Stroke. 1979;10(2):205-207. doi:10.1161/01.STR.10.2.205.

31. Wahab KW. The burden of stroke in Nigeria. Int J Stroke. 2008;3(4):290–292.

32. Walker RW, Rolfe M, Kelly PJ, George MO, James OFW. Mortality and Recovery After Stroke in The Gambia. Stroke. 2003;34(7):1604-1609. doi:10.1161/01.STR.0000077943.63718.67.

33. Walker RW, Jusabani A, Aris E, et al. Post-stroke case fatality within an incident population in rural Tanzania. J Neurol Neurosurg Psychiatry. 2011;82(9):1001-1005. doi:10.1136/jnnp.2010.231944.

34. Law MR, Watt HC, Wald NJ. The underlying risk of death after myocardial infarction in the absence of treatment. Arch Intern Med. 2002;(162):2405-2410.

35. Kannel WB, Feinleib M. Natural History of Angina Pectoris in the Framingham Study. Prognosis and Survival. Am J Cardiol. 1972;(29):154-163.

36. Zukel WJ, Cohen BM, Mattingly TW, Hrubec Z. Survival following first diagnosis of coronary heart disease. Am Heart J. 1969;78(2):159–170.

37. WHO. Scaling up action against NCDs: How much will it cost? http://whqlibdoc.who.int/publications/2011/9789241502313_eng.pdf. Accessed April 7, 2014.

38. Hendriks ME, Bolarinwa OA, Nelissen HE, et al. Costs of cardiovascular disease prevention care and scenarios for cost saving: a micro-costing study from rural Nigeria. J Hypertens. 2015;33(2):376-684. doi:10.1097/HJH.0000000000000402.

39. Gomez GB, Foster N, Brals D, et al. Improving Maternal Care through a State-Wide Health Insurance Program: A Cost and Cost-Effectiveness Study in Rural Nigeria. PLOS ONE. 2015;10(9):e0139048. doi:10.1371/journal.pone.0139048.

40. WHO. WHO | Health service delivery costs. http://www.who.int/choice/cost-effectiveness/inputs/health_service/en/. Published 2008. Accessed October 13, 2014.

41. NHIS. NHIS healthcare providers service price list. http://dhmlnigeria.com/downloads/NHIS_drugs_pricelist_2011.pdf. Published 2011.

42. Adoukonou T, Kouna-Ndouongo P, Codjia J-M, et al. Cout direct hospitalier des accidents vasculaires cérébraux à Parakou au nord du Benin. Pan Afr Med J. 2013;16. doi:10.11604/pamj.2013.16.121.2790.

43. Guinhouya KM, Tall A, Kombate D, et al. [Cost of stroke in Lomé (Togo)]. Sante. 2010. doi:10.1684/san.2010.0192.

44. Gombet TR, Ellenga-Mbolla BF, Ikama MS, Ekoba J, Kimbally-Kaky G. [Cost of emergency cardiovascular care at the University Hospital Center in Brazzaville, Congo]. Med Trop. 2009;69:45-47.

45. Touré K, Ndjaye NM, Sène Diouf F, et al. [Evaluation of the cost of stroke management in Dakar, Senegal]. Med Trop Mars. 2005;65(5):458-464.

46. Kolo P. Cost of managing acute first-ever stroke at the University of Ilorin Teaching Hospital, Nigeria: preliminary report. Unpublished. 2010.

47. Ekwunife OI, Okafor CE, Ezenduka CC, Udeogaranya PO. Cost-utility analysis of antihypertensive medications in Nigeria: a decision analysis. Cost Eff Resour Alloc. 2013;11(1):2.

48. Birabi BN, Oke KI, Dienye PO, Okafor UC. Cost Burden of Post Stroke Condition in Nigeria: A Pilot Study. Glob J Health Sci. 2012;4(6). doi:10.5539/gjhs.v4n6p17.
